# Targeting Mast Cell Activation and MIF‐Mediated Remodelling Enhances Chemotherapy Response in Pancreatic Cancer

**DOI:** 10.1002/advs.202509930

**Published:** 2025-10-29

**Authors:** Libo Wang, Guangcong Shen, Guanpeng Xie, Zekun Li, Xiaoqing Ma, Mengyu Li, Ziyun Liu, Yadi Wang, Zongjing Lv, Qingxiao Fang, Huihui Sun, Ningning Zhao, Chao Yang, Tianxing Zhou, Yongjie Xie, Jun Yu, Jihui Hao

**Affiliations:** ^1^ Pancreas Center Tianjin Medical University Cancer Institute and Hospital National Clinical Research Center for Cancer State Key Laboratory of Druggability Evaluation and Systematic Translational Medicine Tianjin Key Laboratory of Digestive Cancer Tianjin's Clinical Research Center for Cancer Tianjin P. R. China

**Keywords:** cancer‐associated fibroblast, macrophage migration inhibitory factor, neoadjuvant chemotherapy, pancreatic ductal adenocarcinoma, tumor‐associated mast cell

## Abstract

Neoadjuvant gemcitabine plus nab‐paclitaxel (AG) is increasingly applied in pancreatic ductal adenocarcinoma (PDAC), yet its effects on the tumor microenvironment (TME) remain incompletely defined. By integrating eight single‐cell RNA sequencing datasets and nine multicenter transcriptomic cohorts, the dual impact of AG in PDAC is delineated. AG shifts residual malignant cells from basal toward a more indolent classical phenotype and remodels the TME into a more heterogeneous and intricate landscape. Specifically, AG activates tumor‐associated mast cells (TAMCs), reprogrammes myofibroblastic cancer‐associated fibroblasts (myCAFs) into inflammatory CAFs (iCAFs), and enhances suppressive crosstalk between TAMCs, iCAFs, and T cells via the macrophage migration inhibitory factor (MIF) axis. Concurrently, AG reduces exhausted T cells and regulatory T cells while enriching cytotoxic natural killer T cells, reshaping the immune milieu in a manner potentially favorable for immunotherapy. In orthotopic and subcutaneous PDAC models, genetic ablation of TAMCs using Kit^W‐sh^ mice or pharmacologic stabilization using sodium cromoglycate and the MIF antagonist ISO‐1 synergistically improves AG efficacy, with further benefit observed upon addition of anti‐PD‐1 therapy. These findings reveal a previously unrecognized mechanism of AG therapy‐induced immunosuppression and nominate TAMCs‐MIF signaling as a tractable target to optimize neoadjuvant strategies in PDAC.

## Graphical abstract

Neoadjuvant gemcitabine plus nab‐paclitaxel (AG) reprograms pancreatic ductal adenocarcinoma by shifting malignant cells toward a classical phenotype and remodeling the tumor microenvironment. Despite enhancing cytotoxic NKT cells, AG activates tumor‐associated mast cells (TAMCs) and inflammatory CAFs through MIF signaling. Targeting TAMCs or MIF potentiates AG efficacy and synergizes with anti‐PD‐1 therapy, highlighting a promising combination treatment strategy.

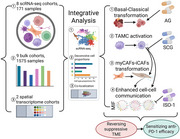

## 1 Introduction

Pancreatic ductal adenocarcinoma (PDAC), one of the most malignant tumors, is characterized by difficulty in early diagnosis, rapid progression, and poor outcome. It is expected to become the second leading cause of cancer‐related death by 2030.^[^ ^1^, ^2^ ^]^ Increasing evidence in recent years suggests that ≈30% of patients are initially diagnosed with locally advanced pancreatic cancer (LAPC), and translational therapy has enabled some patients to regain surgical resection opportunities.^[^ ^3^ ^]^ For LAPC, many guidelines recommend FOLFIRINOX for patients with good performance status (PS, ECOG 0–1) and albumin‐bound paclitaxel (nab‐paclitaxel) combined with gemcitabine (AG) for those with moderate PS score (ECOG 2).^[^ ^2^ ^]^ These are considered the first‐line chemotherapy regimen for PDAC. Multicenter clinical trials have also confirmed that AG followed by FOLFIRINOX sequential treatment can significantly improve the surgical conversion rate and prolong the overall survival (OS) in patients with LAPC having distant metastasis.^[^ ^4^, ^5^ ^]^ However, single‐agent chemotherapy with capecitabine or gemcitabine is generally recommended for patients with poor PS scores.

Despite advances in multimodal strategies, including targeted therapy against KRAS mutations, combinatorial immunotherapy, and radiotherapy following induction chemotherapy, their integration into neoadjuvant therapy for LAPC remains limited.^[^ ^2^, ^3^, ^4^, ^5^ ^]^ Accumulating evidence supports their potential value, yet the current selection of neoadjuvant regimens largely relies on the PS scores of patients, which is inadequate to meet the clinical need for effective local tumor control or meaningful downstaging. Moving forward, a more comprehensive understanding of the tumor microenvironment (TME) alterations induced by various therapeutic regimens is essential. This will facilitate the development of personalized, TME‐informed neoadjuvant strategies and help realize the goal of precision oncology for PDAC, ultimately improving patient outcomes.

The latest evidence substantiates that a neoadjuvant therapy regimen of ≈2 months can increase the 12‐month survival rate from ≈40% to 77%, whereas extending neoadjuvant therapy to roughly 4 months can achieve an 18‐month survival rate of 67% in patients with borderline resectable PDAC.^[^ ^6^ ^]^ Furthermore, our previous study has demonstrated that neoadjuvant therapy followed by surgical resection significantly improves survival for patients with resectable and borderline resectable PDAC, except for patients with stage IA PDAC.^[^ ^7^ ^]^ However, only ≈20–30% of patients ultimately respond favorably to neoadjuvant therapy due to the pronounced tumor heterogeneity, extremely immunosuppressive TME, and abundant stromal components within PDAC.^[^ ^6^ ^]^ Hence, developing targeted combination strategies that can enable precision medicine and ultimately improve the efficacy of neoadjuvant therapy is still challenging.

Multiple mechanisms contribute to resistance to neoadjuvant therapy. For example, recent studies suggest that co‐occurring genetic mutations in *KRAS* and *TP53*, immune tolerance signaling‐mediated T‐cell dysfunction and exclusion from myeloid cells, and pro‐inflammatory polarization of inflammatory cancer‐associated fibroblasts (iCAFs) in the tumor stroma all contribute to enhanced myeloid chemotaxis and chemoresistance.^[^ ^8^ ^]^ Tumor‐associated mast cells (TAMCs) are a pivotal component of tumor‐infiltrating myeloid cells, and their role in regulating angiogenesis and metastasis, promoting the formation of immunosuppressive TME, and serving as a new target for immunotherapy has been increasingly elucidated.^[^ ^9^, ^10^ ^]^ However, the intricate interactions between TAMCs and the TME during neoadjuvant therapy are not fully understood. A deeper elucidation of the role of TAMCs in remodeling the TME in response to neoadjuvant therapy can open new avenues for developing more effective, individualized neoadjuvant therapies.

This study aimed to perform an integrative analysis of eight single‐cell RNA sequencing (scRNA‐seq) datasets to comprehensively characterize microenvironmental alterations in PDAC following two commonly used neoadjuvant therapy regimens: AG and FOLFIRINOX. We specifically focused on elucidating the role of TAMCs in shaping an immunosuppressive TME and mediating chemoresistance after AG. Our findings revealed that AG therapy induced abnormal activation of TAMCs within the TME compared with naive and FOLFIRINOX therapy. These activated TAMCs facilitated the phenotypic transformation of myofibroblastic CAFs (myCAFs) into iCAFs through intercellular communication networks. Simultaneously, TAMCs contributed to T‐cell dysfunction and exhaustion by secreting pro‐inflammatory mediators, including IL‐1 and macrophage migration inhibitory factor (MIF). On the other hand, AG reduced the prevalence of immunosuppressive exhausted T cells and regulatory T cells (Tregs), while promoting the expansion of cytotoxic natural killer T (NKT) cells, thereby establishing a rationale for subsequent combination immunotherapy. We further elucidated that the combination of TAMC stabilizer sodium cromoglycate (SCG) and MIF antagonist ISO‐1 effectively alleviated the immunosuppressive TME and enhanced the chemosensitivity to AG. Notably, this effect was further amplified by incorporation of anti‐PD‐1 therapy, suggesting a promising neoadjuvant strategy for PDAC.

## 2 Results

### 2.1 Single‐Cell Transcriptomic Atlas of PDAC After Neoadjuvant Therapy

**Figure 1:**
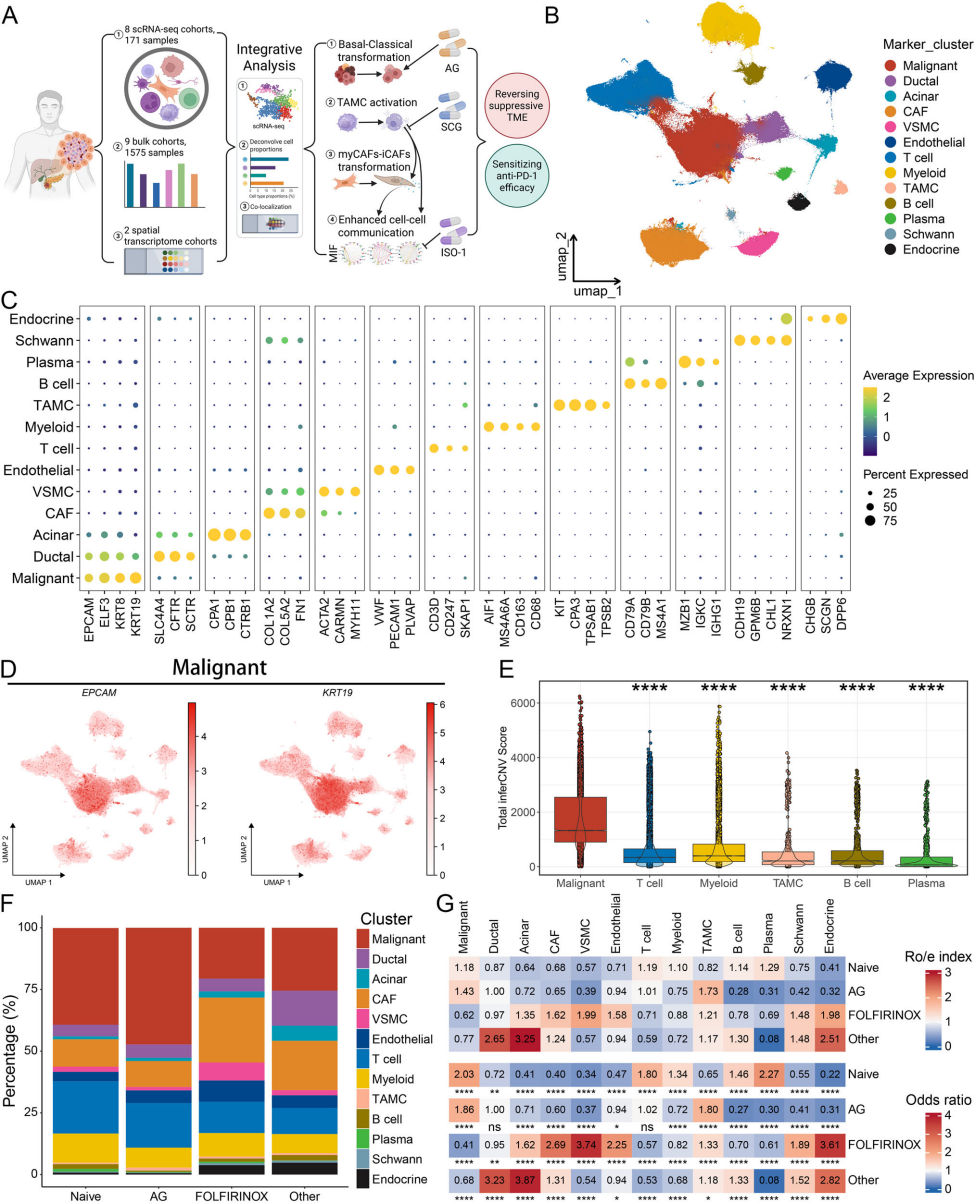
Single‐cell transcriptome landscape of PDAC after treatment with different neoadjuvant regimens. A) The workflow of our study. B) The uniform manifold approximation and projection (UMAP) plot showing the 13 major cell types. Dots represent individual cells, and colors represent different cell populations. C) Dot plot showing the signature markers of these 13 major cell types. D) UMAP plots showing the expression of classical marker *EPCAM* and *KRT19* of malignant cells; the color represents the marker expression value. E) Boxplot showing the total inferred copy number variation (inferCNV) scores in malignant and control cells. F) The percentage of these 13 major cell types in the naive group and treatment groups with different treatment regimens. G) Ro/e index and OR value supporting the preference of these 13 major cell types in different groups. Ro/e denotes the ratio of observed to expected cell number, and OR indicates the odds ratio reflecting tissue‐distribution preferences of cell subsets. Ro/e index > 1 or OR‐value > 1.5 suggests enrichment of the cell subtype in the tissue, while Ro/e index < 1 or OR‐value < 0.5 indicates depletion. ^*^ *P* < 0.05, ^**^ *P* < 0.01, ^***^ *P* < 0.001, ^****^ *P* < 0.0001.

We compiled a PDAC atlas by integrating 171 samples from 8 scRNA‐seq datasets (**Figure** **1**A; Table s1, Supporting Information) to systematically explore the changes in the cellular components of PDAC after neoadjuvant therapies. After correcting for batch effects in various ScRNA‐seq datasets using the Harmony algorithm, we identified 79 distinct clusters in PDAC tissues (Figure S1A–F, Supporting Information). We referred to the representative markers of different clusters supplemented by inferCNV to infer benign and malignant cells, finally annotating them into 13 major cell types (Figure 1B–E; Figures S1G and S2A–L, Supporting Information). Further cell type proportions, Ro/e indexes, and OR values revealed that the infiltration levels of 13 cell types were extremely different after neoadjuvant therapy with various regimens. Overall, malignant cells, T cells, myeloid cells, B cells, and plasma cells were markedly reduced following treatment, whereas normal cells, including ductal, acinar, endocrine cells, as well as stromal components such as CAFs, vascular smooth muscle cells (VSMCs), and endothelial cells were correspondingly increased (Figure s3 A,B, Supporting Information). Notably, the number of malignant cells and TAMCs dramatically increased after AG treatment, whereas the number of interstitial cells, such as CAFs, VSMCs, and endothelial cells, obviously increased after FOLFIRINOX treatment. The infiltrating proportion of normal cells, including ductal, acinar, and endocrine cells, distinctly increased after treatment with other regimens (mainly second‐line treatment regimens such as cetuximab; Figure 1F,G). Furthermore, TAMCs enriched after AG treatment were predominantly localized within primary PDAC tumor tissue but were largely excluded from metastatic lesions (Figure S3C–F, Supporting Information).

### 2.2 Heterogeneity Characteristics of TAMCs After Neoadjuvant Therapy

**Figure 2:**
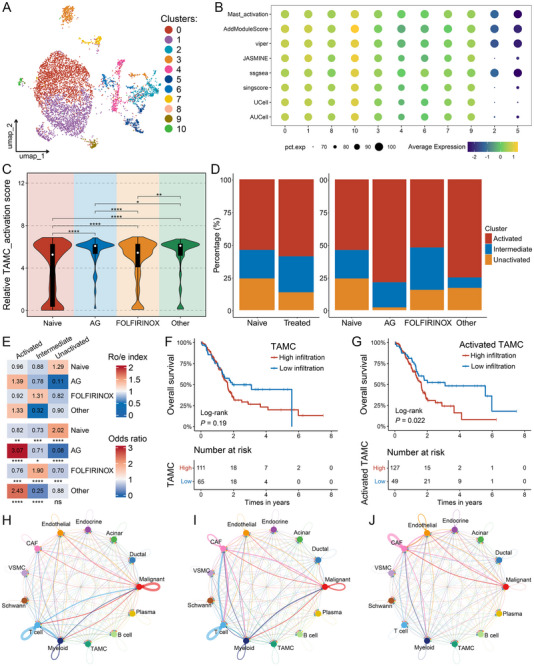
Identification and characterization of TAMC subtypes after treatment with different neoadjuvant regimens. A) The uniform manifold approximation and projection (UMAP) distribution for 11 TAMC clusters from unsupervised clustering. B) Dot plot of TAMC activation scores calculated by seven algorithms as well as summed total activation scores in 11 clusters. C) Violin plot showing activation score levels of TAMCs in the naive group and treatment groups with different treatment regimens. D) The percentage of activated, intermediate, and unactivated TAMCs in the naive group and treated group, as well as in the naive group and treatment groups with different treatment regimens. E) Ro/e index and OR value supporting the preference of the three TAMC subtypes in different groups. Ro/e denotes the ratio of observed to expected cell number, and OR indicates the odds ratio reflecting tissue‐distribution preferences of cell subsets. Ro/e index > 1 or OR‐value > 1.5 suggests enrichment of the cell subtype in the tissue, while Ro/e index < 1 or OR‐value < 0.5 indicates depletion. F, G) Kaplan‐Meier survival curve for overall survival (OS) between groups with high and low estimated proportion of TAMCs (F) and activated TAMCs (G) in the TCGA‐PDAC cohort. H‐J) Cell–cell communication networks among 13 major cell subsets in the naive (H), AG (I), and FOLFIRINOX (J) groups. ^*^ *P* < 0.05, ^**^ *P* < 0.01, ^***^ *P* < 0.001, ^****^ *P* < 0.0001.

The distinct infiltration and heterogeneous activation of TAMCs play a crucial role in immune escape, tumor progression, and individualized therapy.^[^ ^9^, ^11^ ^]^ We used the hallmark activation markers to identify observably activated cluster 0, 1, 8, and 10, intermediate cluster 3, 4, 6, 7, and 9, and other unactivated clusters among the 11 TAMC clusters resulting from unsupervised clustering (**Figure** **2**A,B; Figure S4A–I, Supporting Information). We found that TAMC activation signatures in patients with PDAC after AG neoadjuvant therapy, which were accumulated by seven algorithms, were significantly higher than those observed in other patient groups (Figure 2C). Moreover, the activated TAMC subtype was substantially more abundant in AG and other treatment groups, especially AG group, while the intermediate subtype was obviously increased in FOLFIRINOX (Figure 2D,E). Besides the changes in infiltration abundance, the GSEA analysis at the scRNA‐seq level revealed that TAMCs after AG neoadjuvant therapy exhibited significant enrichment in inflammatory and secretory pathways such as cytokine‐cytokine receptor interaction and humoral immune response (Figure S4J,K, Supporting Information). CellChat analysis also indicated considerable alterations in cell–cell communication between TAMCs and the microenvironment following AG neoadjuvant therapy (Figure 2H–J; Figure S5A–F, Supporting Information). Specifically, the cell–cell interaction between TAMCs and T cells on secretory signals such as CD80, CD86, IFN‐II, IL‐4, and TGF‐β was significantly enhanced in the AG group. The communication between TAMCs and CAFs on inflammatory/immune signals, including CD80, CD86, IL‐1/4, TGF‐β, and VEGF was also considerably elevated (Figure S5A–I, Supporting Information).

Recent studies have established a significant association between TAMCs and poor prognosis in PDAC.^[^ ^9^ ^]^ We used the Bisque deconvolution algorithm^[^ ^12^ ^]^ to estimate the proportions of various cell subsets derived from scRNA‐seq data across three large‐scale cohorts: TCGA‐PDAC (*n* = 176), E‐MTAB‐6134 (*n* = 288), and PDAC_CA_seq (*n* = 182). Kaplan‐Meier survival analysis revealed that elevated TAMC infiltration was significantly correlated with shortened disease‐free survival (DFS) in the TCGA‐PDAC cohort (Figure S6A, Supporting Information). Despite similar trends observed for OS, progression‐free survival (PFS), and disease‐specific survival (DSS), these parameters did not reach statistical significance (Figure 2F; Figure S6B,C, Supporting Information). The presence of activated TAMCs demonstrated a robust and statistically significant association with worse OS, DFS, PFS, and DSS in the TCGA‐PDAC cohort (all *P* < 0.05; Figure 2G; Figure S6D–F, Supporting Information). These findings were further validated in the E‐MTAB‐6134 and PDAC_CA_seq cohorts, where consistent associations were observed for OS and relapse‐free survival (all *P* < 0.05, Figure S6G–J, Supporting Information). Collectively, our results highlighted the promising value of activated TAMCs as independent predictors of dismal prognosis in PDAC.

**Figure 3:**
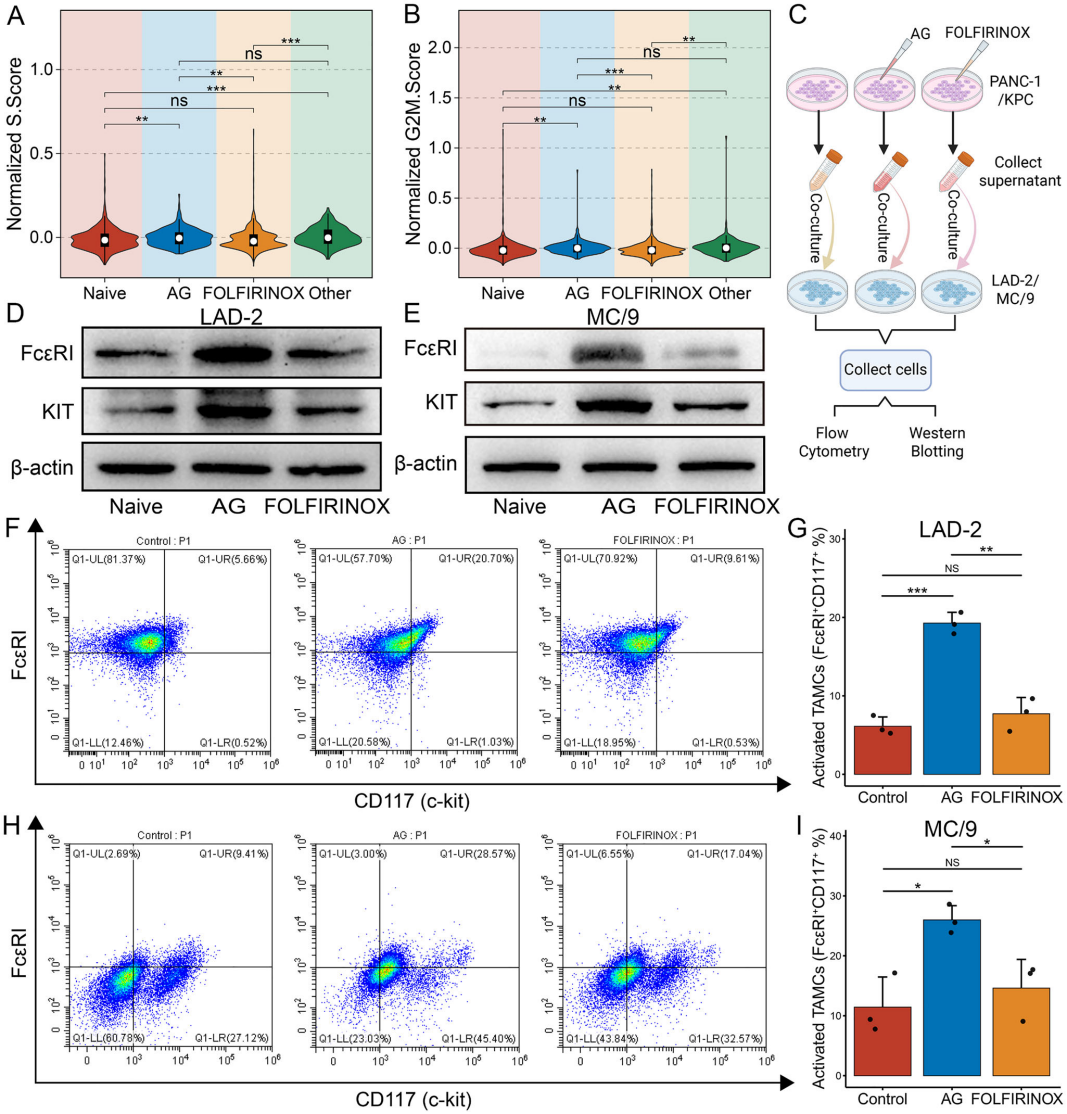
Biological experimental verification of TAMC activation status. A,B) Violin plots showing the S.Score (A) and G2M.Score (B) levels of TAMCs in the naive group and treatment groups with different treatment regimens. C) Schematic representation of tumor cell and mast cell co‐cultures and subsequent mast cell activation assays. D,E) Western blot analysis of human mast cells LAD‐2 (D) and murine mast cells MC/9 (E) stimulated with supernatants derived from tumor cells treated with different treatment regimens. F–I) Flow cytometry analysis of human mast cells LAD‐2 (F,G) and murine mast cells MC/9 (H, I) stimulated with supernatants derived from tumor cells treated with different treatment regimens. Data are from three independent experiments; error bars represent SD; ^*^ *P* < 0.05, ^**^ *P* < 0.01, ^***^ *P* < 0.001, ^****^ *P* < 0.0001.

In addition, the analysis of the TAMC population at the scRNA‐seq level revealed that the AG group exhibited substantially higher cell cycle scores S.Score, and G2M.Score, indicating their relatively active cell cycling and proliferative state (**Figure** **3**A,B). Subsequently, we conducted co‐culture experiments to investigate the effects of various treatment regimens on TAMCs (Figure 3C). The western blot results from human and murine models consistently showed that TAMCs stimulated with the supernatants from AG‐treated tumor cells exhibited elevated expression of TAMC activation markers FcεRI and KIT. In contrast, FOLFIRINOX induced no obvious change in expression (Figure 3D,E). Parallelly, flow cytometry further confirmed that stimulation with the supernatants from AG‐treated tumor cells significantly improved the proportion of activated TAMCs in both human LAD‐2 cells (control: 6.12 ± 1.19%, AG: 19.27 ± 1.37%, FOLFIRINOX: 7.72 ± 2.07%) and murine MC/9 cells (control: 11.44 ± 5.03%, AG: 26.00 ± 2.37%, FOLFIRINOX: 14.62 ± 4.79) (Figure 3F–I). In summary, we identified therapy‐specific TAMC modulation in PDAC. AG treatment, but not the FOLFIRINOX regimen, drove TAMC activation, suggesting new opportunities for microenvironment‐targeted interventions following AG therapy.

### 2.3 Distinct CAF Profiles in PDAC Treated with Various Neoadjuvant Regimens

**Figure 4:**
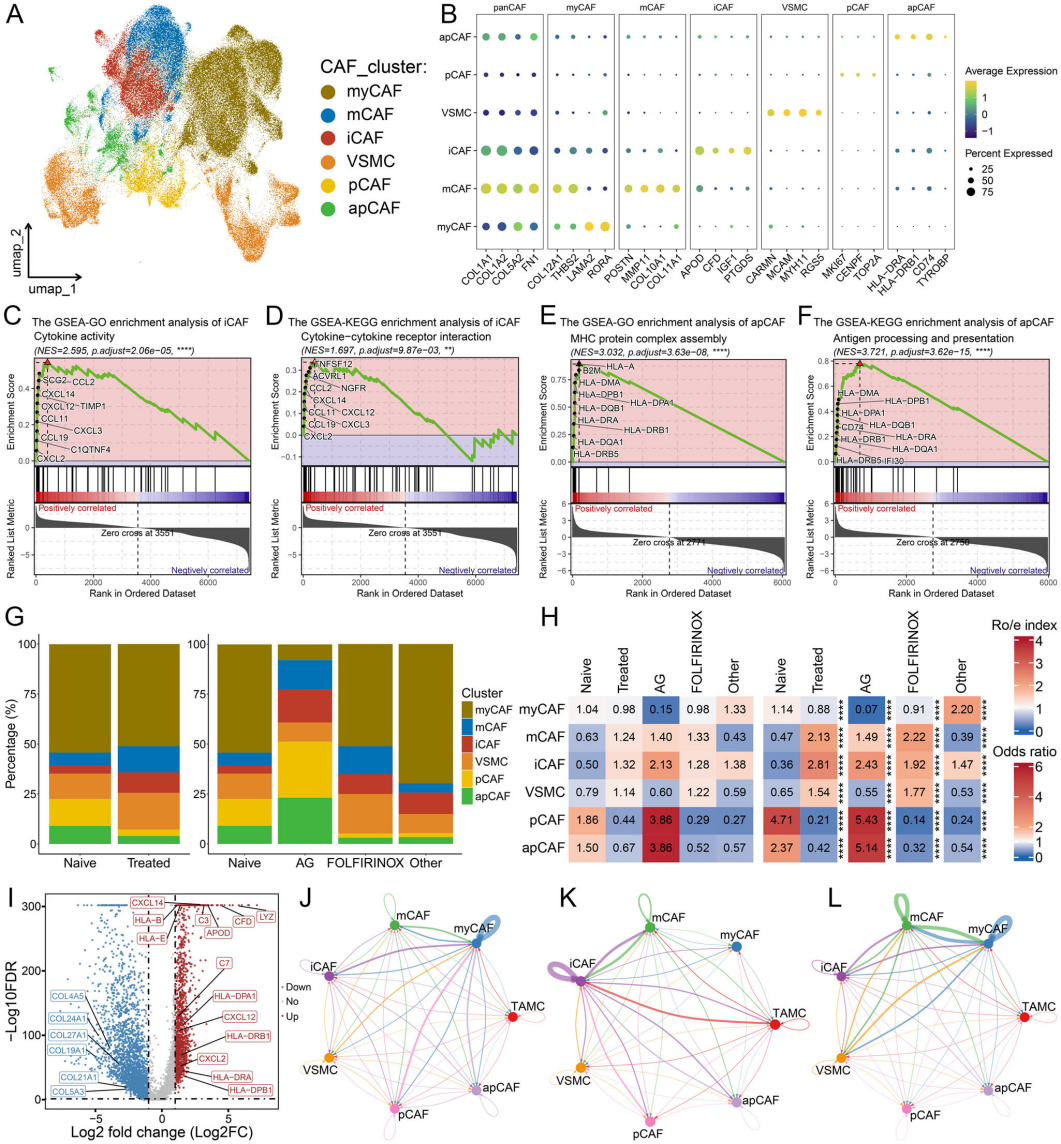
Identification and characterization of CAF subtypes after treatment with different neoadjuvant regimens. A) The uniform manifold approximation and projection (UMAP) plot showing the six cancer‐associated fibroblast (CAF) subtypes. Dots represent individual cells, and colors represent different cell populations. B) Dot plot showing the signature markers of these six CAF subtypes. C, D) Gene set enrichment analysis (GSEA) using the Gene Ontology (GO) and Kyoto Encyclopedia of Genes and Genomes (KEGG) database highlighted the significant biological pathways of iCAFs. E, F) GSEA analysis using the GO and KEGG databases highlighted the significant biological pathways of apCAFs. G) The percentage of six CAF subtypes in the naive group and treated group, as well as in the naive group and treatment groups with different treatment regimens. H) Ro/e index (left panel) and OR value (right panel) supporting the preference of these six CAF subtypes in different groups. Ro/e denotes the ratio of observed to expected cell number, and OR indicates the odds ratio reflecting tissue‐distribution preferences of cell subsets. Ro/e index > 1 or OR‐value > 1.5 suggests enrichment of the cell subtype in the tissue, while Ro/e index < 1 or OR‐value < 0.5 indicates depletion. I) The identification of significantly up‐regulated and down‐regulated differentially expressed genes in CAFs from AG group. J‐L) Cell–cell communication networks between TAMCs and six CAF subtypes in the naive (J), AG (K), and FOLFIRINOX (L) groups. ^*^ *P* < 0.05, ^**^ *P* < 0.01, ^***^ *P* < 0.001, ^****^ *P* < 0.0001.

Accumulating evidence substantiated that CAFs were an indispensable component of the PDAC stroma, playing crucial roles in the high tumor heterogeneity and extremely suppressed TME.^[^ ^13^, ^14^ ^]^ Referring to classical markers and previous findings,^[^ ^14^, ^15^, ^16^ ^]^ we annotated CAF subpopulations resulting from subcluster analysis into six subtypes: myCAFs, matrix CAFs (mCAFs), iCAFs, VSMCs, proliferative CAFs (pCAFs), and antigen‐presenting CAFs (apCAFs) (**Figure** **4**A,B). GO, KEGG, and GSEA enrichment analyses showed that myCAFs were principally associated with fibrosis and matrix sclerosis pathways, such as cell‐matrix adhesion, focal adhesion, and protein localization to cytoskeleton (Figure S7A–D, Supporting Information). Consistent with previous findings,^[^ ^15^, ^16^ ^]^ mCAFs were enriched in extracellular matrix (ECM) remodeling signals, including collagen metabolic process and protein processing in endoplasmic reticulum, besides specifically expressing several ECM remodeling genes (*POSTN*, *MMP11*, *COL10A1*, and *COL11A1*) (Figure S7A,B,E,F, Supporting Information). CFD^+^ CAFs were designated as iCAFs because of the high expression of inflammatory signature genes (*APOD*, *CFD*, and *PTGDS*). They exhibited the highest activation in immune‐related characteristics, including cytokine activity, cytokine‐cytokine receptor interaction, leukocyte migration, and chemotaxis (Figure 4C,D; Figure S7A,B, Supporting Information). CARMN^+^ CAFs were identified as VSMCs due to specific high expression of vascular‐related signatures (*MCAM*, *MYH11*, and *RGS5*) and enrichment in VSMC contraction signals, such as muscle contraction and calcium signaling pathway (Figure S7A,B,G,H, Supporting Information). In addition, we identified a cluster of CAFs that highly expressed cell cycle‐related markers (*MKI67*, *CENPF*, and *TOP2A*) and were characterized by proliferative biological features such as positive regulation of cell cycle, DNA replication, and P53 signaling pathway, which was consistent with reported pCAFs (Figure S7A,B,I,J, Supporting Information).^[^ ^14^, ^15^, ^16^, ^17^, ^18^ ^]^ Finally, CD74^+^ CAFs were classified as apCAFs with higher expression of antigen presentation signature (*HLA‐DRA*, *HLA‐DRB1*, and *CD74*) and enriched in MHC protein complex assembly and antigen processing and presentation pathways (Figure 4E,F; Figure S7A,B, Supporting Information).

We further explored the presence and characteristics of six CAF subtypes among different groups. The mCAFs and iCAFs were highly enriched in treated patients, particularly those treated with AG (Figure 4G,H). The number of apCAFs was significantly reduced in patients treated with FOLFIRINOX and other regimens, but it distinctly increased in the AG group compared with the naive group (Figure 4G,H). Furthermore, the expression levels of several iCAF markers, such as *CFD*, *C3*, and *APOD*, as well as those of apCAFs, including *HLA‐B*, *LYZ*, *HLA‐DPA1*, *HLA‐DRA*, and *HLA‐E*, were significantly elevated. In contrast, the expression levels of several collagens representing myCAFs, such as *COL4A5*, *COL19A1*, and *COL24A1*, significantly decreased in CAFs from the AG group. This confirmed that neoadjuvant therapy with the AG regimen led to a transformation of CAFs within the TME from a stromal phenotype to an inflammatory chemotaxis and immunophenotype (Figure 4I). Overall, these findings emphasize the potential significance of CAF subtype transformation in PDAC following AG treatment.

### 2.4 Activated TAMCs Promoted CAF Subtype Transformation Following AG Neoadjuvant Therapy

**Figure 5:**
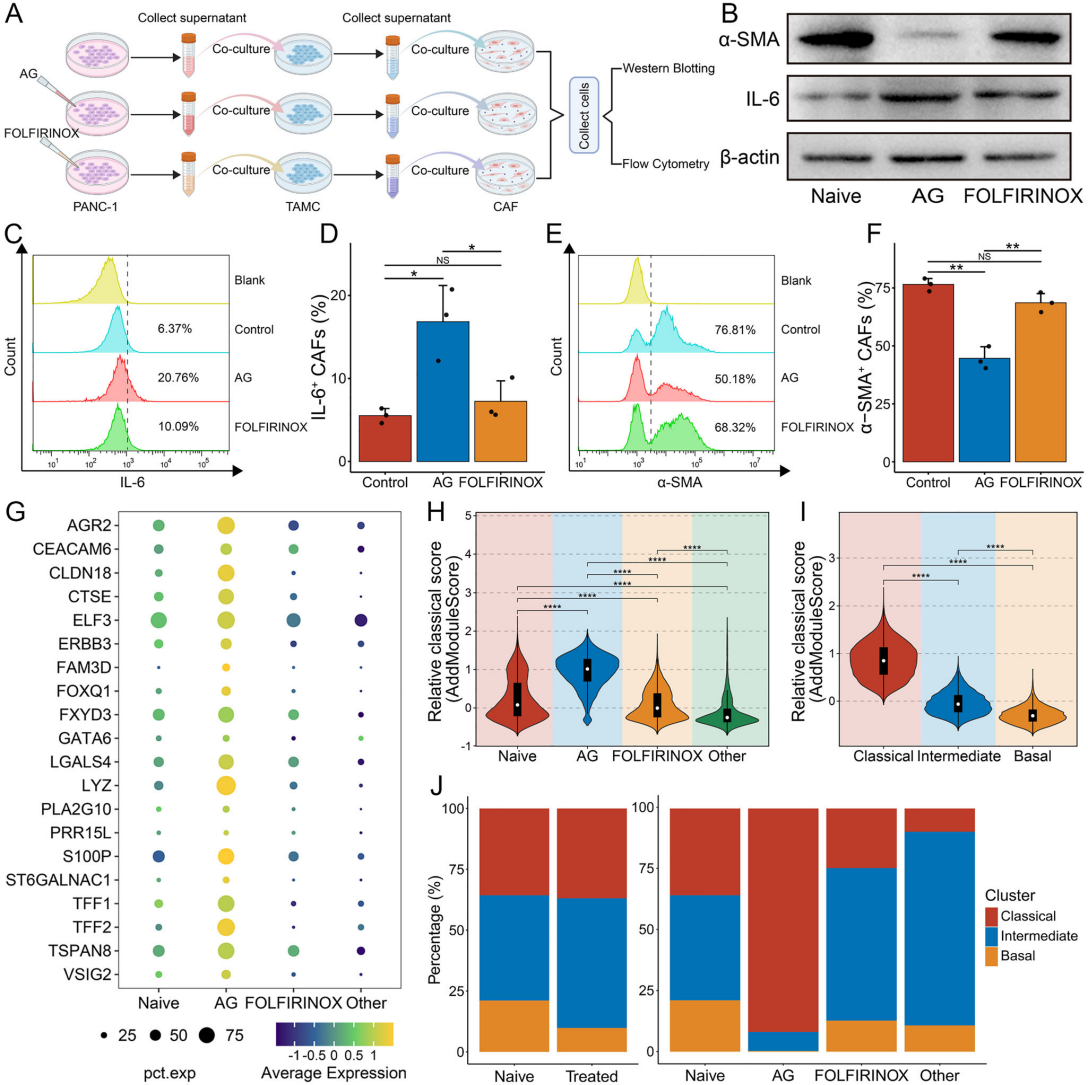
Biological experimental validation of CAF signature and molecular characterization of tumor cells. A) Schematic representation of tumor cells, TAMCs, and cancer‐associated fibroblasts (CAFs) co‐cultures and subsequent CAFs status detection assays. B) Western blot analysis of primary CAFs stimulated with mast cells‐conditioned media treated with different treatment regimens. C, D) The percentage of IL‐6 expression in primary CAFs stimulated with mast cells‐conditioned media treated with blank control, AG, and FOLFIRINOX groups was detected and analyzed by flow cytometry. E, F) The percentage of α‐SMA expression in primary CAFs stimulated with mast cells‐conditioned media treated with blank control, AG, and FOLFIRINOX groups was detected and analyzed by flow cytometry. Data are from three independent experiments; error bars represent SD. G) Dot plots showing the mean expression levels of classical markers in tumor cells from the four groups of patients. H) Violin plots showing classical signature scores calculated by AddModuleScore function in tumor cells from four groups of patients. I) Violin plots showing classical signature scores calculated by AddModuleScore function in tumor cells from three malignant cell lineage subtypes. J) The percentage of three malignant cell lineage subtypes in the naive group and treated group, as well as in the naive group and treatment groups with different treatment regimens. ^*^ *P* < 0.05, ^**^ *P* < 0.01, ^***^ *P* < 0.001, ^****^ *P* < 0.0001.

We conducted cell–cell communication analysis in combination with TAMCs, whose numbers significantly increased after AG therapy, to gain insights into the impact of CAF subtype transformation caused by AG neoadjuvant therapy on the TME at the single‐cell level. Utilizing the CellChat algorithm, we identified observably enhanced interactions on inflammatory pathways (TGF‐β, IL‐4, and TRAIL) between TAMCs and CAFs in the AG group compared with both naive and FOLFIRINOX groups, particularly between TAMCs and iCAFs (Figure 4J–L; Figure S8A–I, Supporting Information). Based on our observation that the supernatant derived from AG‐stimulated PDAC cells activated TAMCs (Figure 3), we then performed further co‐culture experiments. Briefly, we stimulated TAMCs using tumor cell culture supernatants treated with different regimens and then co‐cultured them with primary CAFs using the aforementioned TAMC‐conditioned media (**Figure** **5**A). TAMC‐conditioned media from AG‐stimulated supernatants, but not from FOLFIRINOX‐stimulated supernatants, resulted in a decrease in the expression of myCAF marker α‐SMA and an increase in the expression of iCAF marker IL‐6 in primary CAFs compared with placebo controls (Figure 5B). Flow cytometry further revealed that TAMC‐conditioned media derived from AG‐stimulated tumor cell supernatants enhanced the iCAFs phenotype and attenuated its myCAFs phenotype in primary CAFs, as evidenced by a noticeable increase in the proportion of IL‐6^+^ CAFs (control: 5.52 ± 0.86%, AG: 16.85 ± 4.36%, FOLFIRINOX: 7.24 ± 2.48%) and a distinct decrease in the proportion of α‐SMA^+^ CAFs (control: 76.54 ± 2.54%, AG: 44.65 ± 4.99%, FOLFIRINOX: 68.61 ± 3.97%) (Figure 5C–F). Collectively, these results confirmed the potential possibility that activated TAMCs drove myCAFs‐to‐iCAFs subtype transformation through some inflammatory mediators following AG neoadjuvant therapy, thereby reshaping the stromal microenvironment.

### 2.5 AG Neoadjuvant Therapy Induced Basal‐to‐Classical Subtype Switch in PDAC

The progress of precision oncology has led to the emergence of an increasing number of molecular subtyping of PDAC based on next‐generation sequencing. Among them, Moffitt's classification, which divides PDAC into basal and classical subtypes, has been widely recognized.^[^ ^19^, ^20^, ^21^ ^]^ Using previously established markers,^[^ ^19^, ^22^, ^23^ ^]^ we found that classical subtype markers were broadly upregulated, whereas basal subtype markers were downregulated in patients with PDAC receiving AG neoadjuvant therapy (Figure 5G,H; Figure S9A,B and Table S4, Supporting Information). In addition, our findings validated the classification framework proposed by Pei et al., which reliably stratifies PDAC cells into classical, intermediate, and basal lineages (Figure 5I; Figure S9C,D, Supporting Information). Strikingly, analyses of percentage histograms, Ro/e indexes, and OR values consistently demonstrated that AG treatment decreased the basal population while significantly expanding the classical subtype, whereas FOLFIRINOX and Other regimens predominantly induced intermediate subtypes (Figure 5J; Figure S9E, Supporting Information).

**Figure 6:**
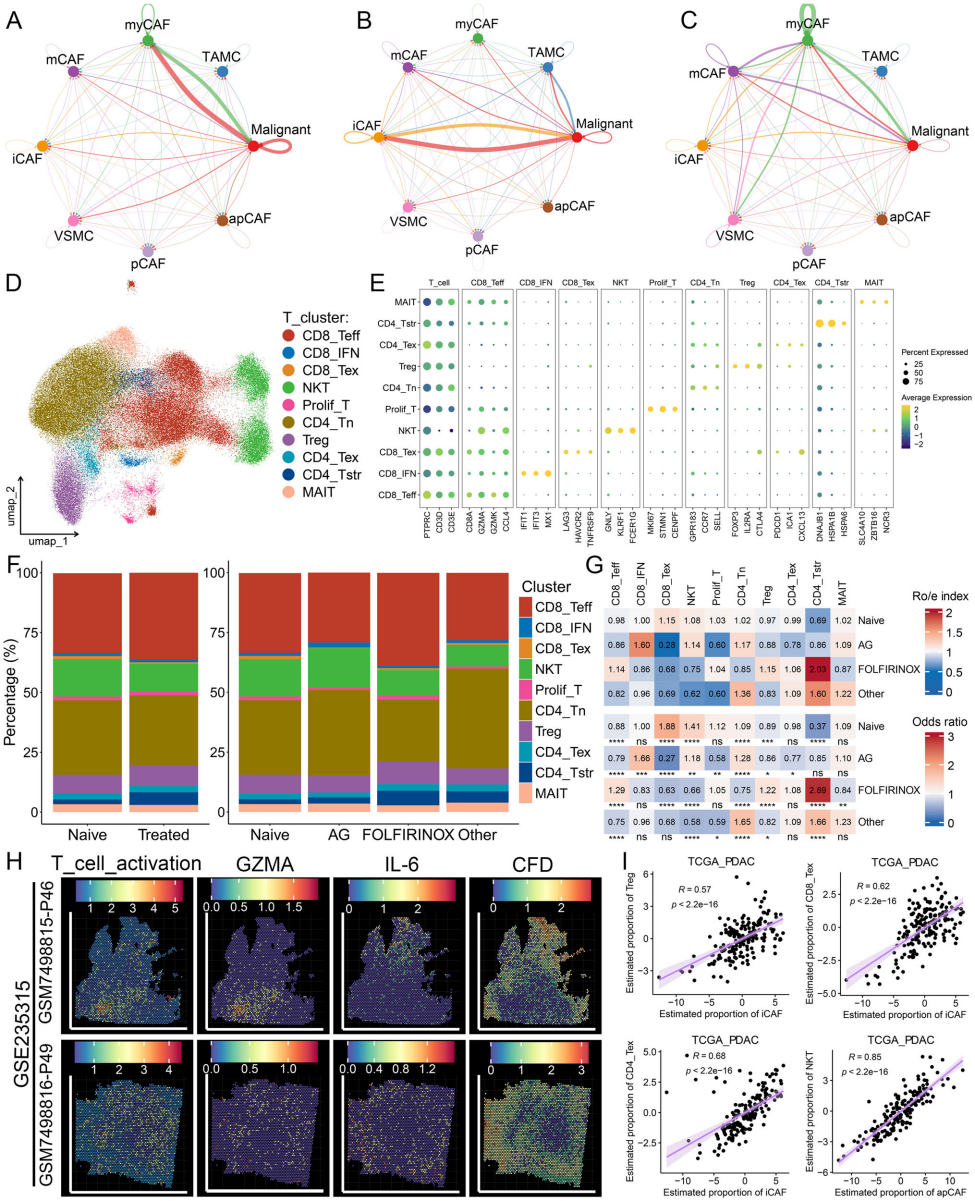
Identification and characterization of T‐cell subtypes after treatment with different neoadjuvant regimens. A–C) Cell–cell communication networks among TAMCs, tumor cells, and six CAF subtypes in the naive (A), AG (B), and FOLFIRINOX (C) groups. D) The uniform manifold approximation and projection (UMAP) plot showing the 10 T‐cell subtypes. Dots represent individual cells, and colors represent different cell populations. E) Dot plot showing the signature markers of these 10 T‐cell subtypes. F) The percentage of 10 T‐cell subtypes in the naive group and treated group, as well as in the naive group and treatment groups with different treatment regimens. G) Ro/e index and OR value supporting the preference of these 10 T‐cell subtypes in different groups. Ro/e denotes the ratio of observed to expected cell number, and OR indicates the odds ratio reflecting tissue‐distribution preferences of cell subsets. Ro/e index > 1 or OR‐value > 1.5 suggests enrichment of the cell subtype in the tissue, while Ro/e index < 1 or OR‐value < 0.5 indicates depletion. H) Spatial feature plots of the seven algorithms T‐cell activation score, T‐cell activation marker GZMA, as well as inflammatory cancer‐associated fibroblasts (iCAFs) markers IL‐6 and CFD in P46 and P49 tissue sections (from left to right, GSE235315). I) The correlations between the estimated proportions by Bisque deconvolution algorithm of iCAFs and regulatory T cells (Tregs), CD8^+^ exhausted T cells (CD8_Tex), and CD4^+^ exhausted T cells (CD4_Tex), as well as antigen‐presenting CAFs (apCAFs) and natural killer T (NKT) cells in the TCGA‐PDAC cohort. ^*^ *P* < 0.05, ^**^ *P* < 0.01, ^***^ *P* < 0.001, ^****^ *P* < 0.0001.

Further cell–cell communication analysis revealed markedly enhanced interactions among TAMCs, iCAFs, and tumor cells following AG neoadjuvant therapy (**Figure** **6**A–C). Specifically, FOLFIRINOX exhibited minimal perturbation of the activated bidirectional signaling networks observed following the AG regimen, involving TAMC‐derived ligands (e.g., DHEA, EGF, GDNF, NGF, NTF3/4/5, RELN, SEMA4D, TGFB1/2/3), iCAF‐secreted factors (e.g., ITGAV_ITGB1, BMP2/4, CDH2/3, COMP, IL‐2/7/13/15, SEMA3C, SEMA4A, THBS4, THY1, and TNFSF12), and cognate tumor cell surface receptors (Figures S10A–F, Supporting Information). Collectively, these results suggested that the intercellular crosstalk between tumor cells, TAMCs, and iCAFs within the TME was enhanced after AG neoadjuvant therapy.

### 2.6 TAMCs and iCAFs‐Driven Immunosuppressive Microenvironment Remodeling Following AG Neoadjuvant Therapy

Considering the crucial role of TAMCs and iCAFs in shaping TME,^[^ ^9^, ^15^, ^24^, ^25^ ^]^ we next investigated whether the enrichment of these cells after AG neoadjuvant therapy could reprogram the TME in PDAC. Referring to the well‐established T‐cell markers from the TCellAtlas database and previous studies,^[^ ^26^ ^]^ we annotated T cells in PDAC into 10 subtypes (Figure 6D,E). Similar to previous findings,^[^ ^27^ ^]^ the overall composition of T‐cell subtypes was comparable between naive and treated groups. Notably, the proportion of CD8_IFN cells, characterized by elevated expression of interferon‐stimulated genes (IFIT1, IFIT3, and MX1), as well as natural killer T (NKT) cells, substantially increased following AG neoadjuvant therapy. This shift is accompanied by a concomitant reduction in immunosuppressive populations, including exhausted CD8^+^ T cell (CD8_Tex), regulatory T cell (Treg), and exhausted CD4^+^ T cell (CD4_Tex) (Figure 6F,G). These findings indicated that AG treatment might shape an immunogenic antitumor TME and also provide a rationale for subsequent immunotherapy.

**Figure 7:**
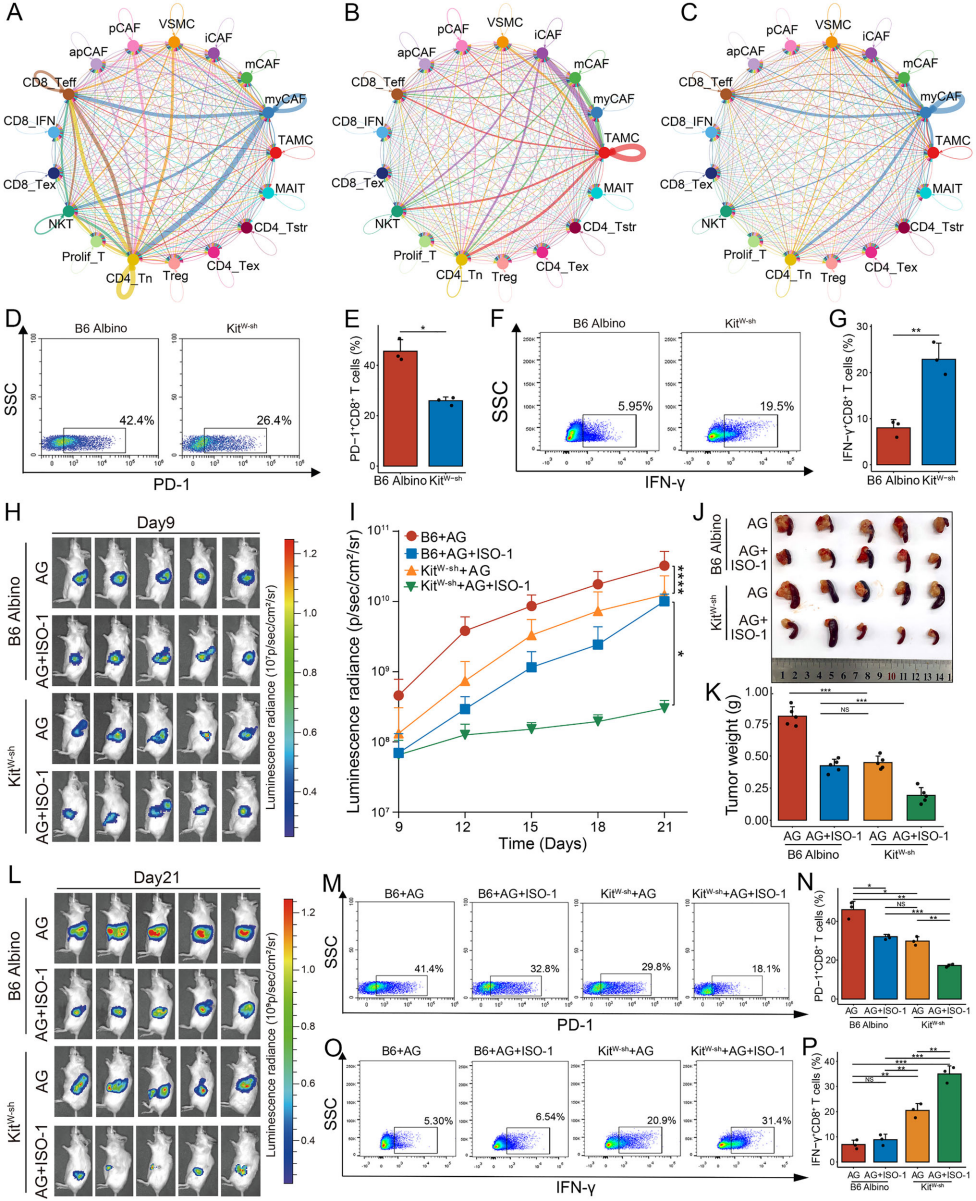
Targeting mast cells and MIF signaling reverses the immunosuppressive microenvironment and enhances sensitivity to AG in preclinical models. A‐C) Cell–cell communication networks among TAMCs, six CAF subtypes, and 10 T‐cell subsets in the naive (A), AG (B), and FOLFIRINOX (C) groups. D‐G) Flow cytometry analysis of B6 Albino and Kit^W‐sh^ genetically engineered mice showing the proportions of PD‐1^+^ exhausted CD8^+^ T cells (D, E), and IFN‐γ⁺ effector CD8^+^ T cells (F, G). H, L) Bioluminescent images at days 9 (H) and 21 (L) following AG or AG combined with MIF antagonist (ISO‐1) treatment in orthotopic xenograft models established in B6 Albino and Kit^W‐sh^ mice. I) Longitudinal bioluminescence intensity curves from day 9 to day 21 in orthotopic tumor‐bearing mice across four treatment groups. J, K) Representative tumor images (J) and quantified tumor weights (K) at the endpoint of treatment across four experimental groups. M‐P) Flow cytometry analysis of orthotopic tumors harvested from four treatment groups showing the proportions of PD‐1^+^ exhausted CD8^+^ T cells (M, N) and IFN‐γ⁺ effector CD8^+^ T cells (O, P). The FC data are from three independent experiments; error bars represent SD; ^*^ *P* < 0.05, ^**^ *P* < 0.01, ^***^ *P* < 0.001, ^****^ *P* < 0.0001.

In contrast, the spatial transcriptomics from two independent studies demonstrated that iCAFs mediated cytotoxic T‐cell exclusion from tumor niches in PDAC tissues (Figure 6H; Figure S11A, Supporting Information). Using the Bisque deconvolution algorithm, we observed strong positive correlations among iCAFs and Tregs, CD8_Tex as well as CD4_Tex cells across five independent multicenter transcriptomic cohorts (Figure 6I; Figure S11B–S, Supporting Information). As anticipated, apCAFs were significantly positively correlated with effector CD8⁺ T (CD8_Teff) and NKT cells (Figure 6I; Figure S11B–S, Supporting Information). Enhanced cellular crosstalk was observed among TAMCs, iCAFs, and T cells through signaling pathways such as COLLAGEN, EPHA, IL‐1, and MIF, compared with that in the naive and FOLFIRINOX groups, suggesting their potential role in shaping immunosuppressive TME following AG neoadjuvant therapy (**Figure** **7**A–C; Figure S12, Supporting Information). Notably, the MIF pathway, recognized as a promising therapeutic target, demonstrates distinctly enhanced interactions between TAMCs as well as AG treatment‐induced increased mCAFs, iCAFs, and apCAFs with target T‐cell subtypes, including NKT cells and naive CD4^+^ T (CD4_Tn) cells (Figure S12J–L, Supporting Information). This suggests that the MIF pathway may contribute to therapy‐specific microenvironment remodeling after AG neoadjuvant therapy.

Based on these findings, we next employed genetically engineered mouse models to investigate whether enriched TAMCs following AG treatment could reprogram the immunosuppressive TME in vivo. Mast cell‐deficient Kit^W‐sh^ mice and B6 Albino mice were used to establish xenograft models by subcutaneously injecting 1.5 × 10^6^ KPC cells. After 21 days, the xenografts were harvested, and the immune status was assessed by flow cytometry. Kit^W‐sh^ mice exhibited markedly reduced proportions of PD‐1^+^ and TIM‐3⁺ exhausted T cells besides substantially increased infiltration of effector cells such as IFN‐γ⁺, TNF‐α⁺, and GZMB⁺ T cells (Figure 7D–G; Figure S13A–F, Supporting Information). These findings demonstrated that TAMC deletion alleviated their suppressive effect on T cells.

### 2.7 Targeting Mast Cells and MIF Synergistically Sensitized PDAC to AG Treatment

The growing understanding of the multifaceted roles of MIF in shaping the immunosuppressive microenvironment, malignant progression, and metastasis has stimulated the development of targeted approaches, ranging from small‐molecule inhibitors and monoclonal antibodies.^[^ ^28^, ^29^, ^30^ ^]^ The results from four large multicenter cohorts indicated significant overexpression of MIF in PDAC tumor tissues (Figure S13G–J, Supporting Information). Moreover, scRNA‐seq data also revealed upregulated expression of MIF in CAFs after AG neoadjuvant therapy (Figure S13K, Supporting Information). These findings collectively established a rationale for targeted MIF in therapeutic development. Subsequently, we conducted in vivo experiments to investigate combination therapy strategies for PDAC.

We next verified whether ablating TAMCs or targeting the MIF‐mediated crosstalk among TAMCs, iCAFs, and T cells under the background of AG neoadjuvant therapy could ameliorate the suppressive TME and its pro‐tumor effects. The luciferase‐labeled KPC cells were orthotopically injected into the pancreas of both B6 Albino and Kit^W‐sh^ mice, and the mice were randomly divided into two experimental groups. Three days later, gemcitabine and nab‐paclitaxel (gemcitabine 25 mg kg^−1^ plus nab‐paclitaxel 15 mg kg^−1^) were injected intraperitoneally every week in four groups (B6 + AG, B6 + AG + ISO‐1, Kit^W‐sh^ + AG, Kit^W‐sh^ + AG + ISO‐1), whereas the mice in the ISO‐1 treatment groups received an additional intraperitoneal injection of ISO‐1 (10 mg kg^−1^) every day.^[^ ^31^ ^]^ Longitudinal IVIS imaging from day 9 onward exhibited that TAMC ablation and MIF antagonist (ISO‐1) significantly attenuated tumor burden in the AG setting, with the combination regimen exhibiting supra‐additive therapeutic effects suggestive synergistic mechanisms (Figure 7H,L). Furthermore, the flow cytometry results also revealed that ISO‐1 significantly reduced the proportions of PD‐1^+^ and TIM‐3^+^ exhausted T cells while increasing IFN‐γ⁺, TNF‐α⁺, and GZMB⁺ effector T‐cell populations, which was markedly enhanced after combined TAMC ablation (Figure 7M–P; Figure S13L–Q, Supporting Information). Overall, these findings suggested that MIF antagonist ISO‐1 combined with TAMC ablation could relieve immunosuppressive TME and sensitize AG efficacy in preclinical PDAC models.

**Figure 8:**
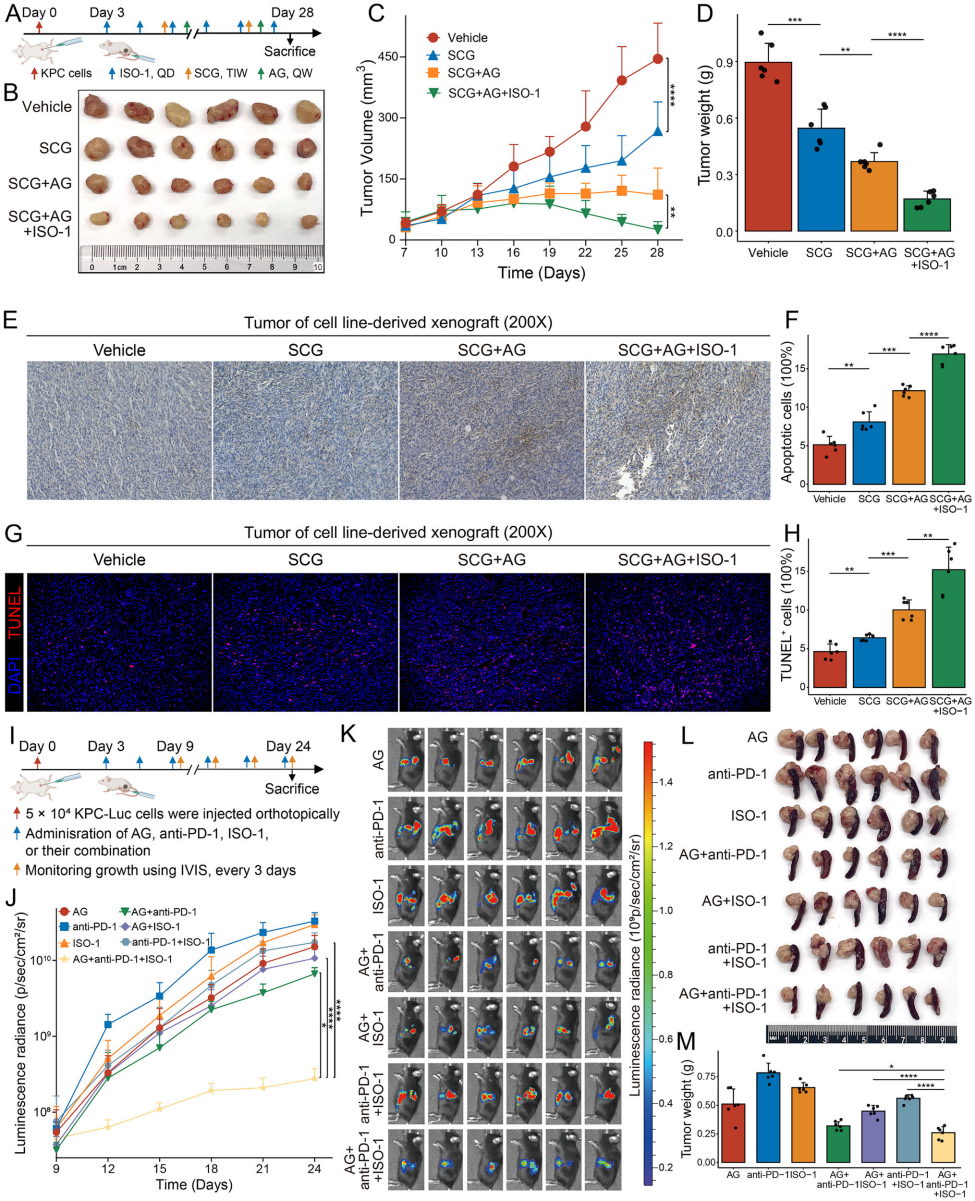
Pharmacological inhibition of mast cells and MIF signaling enhances sensitivity to AG and anti‐PD‐1 immunotherapy in preclinical models. A) Schematic outline of the subcutaneous tumor model used to evaluate the antitumor effects of the mast cell stabilizer sodium cromoglycate (SCG) in combination with the MIF antagonist ISO‐1. A total of 1.5 × 10⁶ KPC cells were subcutaneously injected into C57BL/6 mice. Beginning on day 3 post‐inoculation, mice received vehicle, SCG (75 mg kg^−1^, i.p., three times weekly), ISO‐1 (10 mg kg^−1^, i.p., daily), or AG therapy (gemcitabine 25 mg kg^−1^ plus nab‐paclitaxel 15 mg kg^−1^, i.p., weekly). Tumor diameters were measured every three days starting on day 7 using calipers, and tumors were harvested on day 28. B–D) Representative tumor images (B), longitudinal tumor growth curves from day 7 to 28 (C), and endpoint tumor weights (D) across the four experimental groups. E–H) Immunohistochemical staining for cleaved caspase‐3 (E, F) and TUNEL staining (G, H) in tumors from the four treatment groups, indicating the proportions of apoptotic cells. I) Schematic outline of the orthotopic xenograft model used to evaluate the sensitizing effect of ISO‐1 on anti‐PD‐1 immunotherapy in the context of AG neoadjuvant therapy. A total of 5 × 10^4^ luciferase‐labeled KPC cells were orthotopically injected into the pancreas of C57BL/6 mice. Starting on day 3 post‐inoculation, mice were treated with AG, anti‐PD‐1 antibody (10 mg kg^−1^, i.p., every three days), ISO‐1, or their combination. Tumor burden was monitored every three days using a small‐animal in vivo imaging system beginning on day 9, and tumors were harvested on day 24. J) Longitudinal bioluminescence intensity curves from day 9 to 24 in orthotopic tumor‐bearing mice across seven treatment groups. K) Bioluminescent images at day 24 of orthotopic xenograft tumors in the seven experimental groups. L, M) Representative tumor images (L) and endpoint tumor weights (M) across the seven experimental groups. ^*^ *P* < 0.05, ^**^ *P* < 0.01, ^***^ *P* < 0.001, ^****^ *P* < 0.0001.

SCG has been largely explored for its role in inhibiting TAMC degranulation and cytokine release.^[^ ^9^, ^32^ ^]^ The antitumor efficacy of SCG plus ISO‐1 in the context of AG therapy in vivo was evaluated by establishing subcutaneous KPC cell‐derived xenograft models in C57BL/6 mice, which were then randomly allocated into four groups. Pharmacological interventions were initiated on day 3 post‐implantation as follows: SCG, ISO‐1, and AG therapy. The tumor diameters were measured every 3 days beginning on day 7 until the xenografts were harvested on day 28 (**Figure** **8**A). Consistent with the orthotopic model, SCG monotherapy significantly inhibited tumor growth and reduced tumor burden. In contrast, the combination of SCG and AG/ISO‐1 had remarkable effects compared with SCG alone or SCG plus AG regimens (Figure 8B–D). Consistent with tumor growth inhibition, tumors from the SCG and SCG combined with AG treatment groups exhibited increased numbers of apoptotic cells, as evidenced by cleaved caspase‐3 and TUNEL staining. This effect was further enhanced by the addition of ISO‐1 (Figure 8E–H). These findings highlighted the therapeutic potential of targeting TAMCs and their MIF‐mediated crosstalk with iCAFs and T cells to sensitize PDAC to AG therapy.

### 2.8 Targeting Enhanced MIF Signaling Potentiates Anti‐PD‐1 Immunotherapy Efficacy in the Neoadjuvant AG Neoadjuvant Therapy Setting

To directly evaluate the impact of targeting MIF signaling on immunotherapy efficacy under AG treatment, luciferase‐labeled KPC cells were orthotopically implanted into the pancreas of C57BL/6 mice, which were then randomized into seven treatment groups. Beginning on day 3 post‐inoculation, mice received AG, anti‐PD‐1 antibody (10 mg kg^−1^, i.p., every three days), ISO‐1, or their combinations. Tumor growth was monitored via bioluminescence imaging every three days starting on day 9, and all tumors were harvested on day 24 (Figure 8I). Longitudinal imaging revealed that ISO‐1 combined with anti‐PD‐1 therapy significantly reduced tumor burden in the setting of AG treatment (Figure 8J–M). Together, these results demonstrate that inhibition of MIF signaling sensitizes PDAC to anti‐PD‐1 immunotherapy in the context of AG neoadjuvant therapy.

## 3 Discussion

TAMCs play a dual role in cancer. They accelerate tumor invasion and metastasis by promoting angiogenesis and destroying the ECM, as well as by recruiting immunosuppressive cells such as Tregs to promote tumor immune escape. Additionally, they orchestrate antitumor immunity in specific contexts and strengthen the effect of immunotherapy by activating cytotoxic T cells and B cells.^[^ ^9^, ^10^, ^11^, ^24^, ^32^, ^33^, ^34^, ^35^ ^]^ Recent advances in precision oncology have led to more patients with LAPC undergoing surgical resection after transformation with neoadjuvant therapy.^[^ ^3^ ^]^ However, the functional heterogeneity and possible intervention strategies of TAMCs in PDAC populations receiving various neoadjuvant regimens remain unexplored. Our study confirmed that AG neoadjuvant therapy induced TAMC activation, subsequently driving immunosuppressive TME remodeling by promoting the transformation of myCAFs into iCAFs and enhancing crosstalk with other cellular components. Targeting TAMCs and disrupting the MIF‐mediated signaling interaction among TAMCs, iCAFs, and T cells substantially revitalized the immunosuppressive TME and synergistically sensitized AG chemosensitivity.

Recent studies have identified the presence of stem cell factor (SCF) in PDAC cells. SCF is a key mediator involved in TAMC proliferation, activation, and chemotaxis, contributing to TAMC chemotaxis and recruitment.^[^ ^9^, ^33^, ^36^ ^]^ Single‐cell level analysis revealed a distinct increase in activated TAMCs after AG treatment, accompanied by a transition to secretory phenotypes. Combined with the multicenter cohorts, we further underscored that activated TAMCs, rather than the total TAMC population, were significantly associated with worse PDAC prognosis. These findings extend previous findings of a correlation between total TAMC infiltration and poor PDAC prognosis.^[^ ^9^ ^]^ Moreover, co‐culture experiments involving tumor cells and TAMCs following different treatment regimens provided strong evidence supporting our hypothesis: AG‐treated PDAC cells significantly promoted TAMC activation. The crucial role of numerous mediators, such as SCF, in this process needs further exploration.

On the other hand, AG neoadjuvant therapy created a dynamic TME feedback loop: it activated TAMCs, which in turn remodeled the PDAC stroma by converting myCAFs into an immunosuppressive phenotype iCAFs, thus revealing a novel mechanism of microenvironment‐mediated therapeutic resistance. It has been well established that TAMCs can stimulate the proliferation of CAFs through secreting IL‐13 and tryptase.^[^ ^36^ ^]^ Building upon this foundation, our study identified and validated a novel finding: TAMC activation after AG neoadjuvant therapy induced CAFs to shift the phenotype from myCAFs, which are involved in ECM remodeling and then promoting tumor invasion, metastasis, and therapeutic resistance, to the iCAFs, which exert pro‐inflammatory polarization and interfere with the TME by secreting inflammatory mediators. In conclusion, our findings emphasized that AG neoadjuvant therapy‐induced TAMC activation and subsequent transformation of myCAFs to iCAFs exacerbated the immunosuppressive TME in PDAC, besides guiding the direction for the subsequent development of therapeutic targets and combination therapeutic strategies.

Emerging evidence has confirmed that basal PDAC possesses more advanced grade, higher frequencies of *TP53*/*KRAS* driver mutations, worse prognosis, and poorer responsiveness to FOLFIRINOX. FOLFIRINOX treatment induced the evolution of PDAC to the mesenchymal phenotype (highly correlated with the basal subtype).^[^ ^19^, ^21^, ^23^, ^37^ ^]^ Our study revealed that AG treatment drove patients with PDAC from basal subtype to classical subtype transformation, underscoring the positive significance of AG as a neoadjuvant therapy to some extent.

A previous scRNA‐seq study on eight patients with PDAC who underwent surgical resection following neoadjuvant AG therapy revealed that AG therapy fostered an immunogenic antitumor microenvironment.^[^ ^27^ ^]^ In addition, a spatial transcriptomic analysis integrating matched primary and multiple metastatic lesions from 13 patients with PDAC showed that peritumoral CAF‐derived CXCL12 established an immune‐excluded phenotype by physically preventing fetal effector cells, such as T cells and plasma cells, from infiltrating the tumor core region.^[^ ^22^ ^]^ In our study, spatial mapping further demonstrated mutually exclusive distribution patterns between iCAFs and cytotoxic T lymphocytes within the tumor interior. These findings indicated that although AG treatment might modestly increase the infiltration of effector T cells, the persistent presence of immunosuppressive stromal components such as iCAFs might spatially exclude these cells from the tumor core, thereby limiting their antitumor efficacy. Moreover, using deconvolution algorithms, we also observed a significant positive correlation between iCAF abundance and the infiltration of Tregs, CD8_Tex, and CD4_Tex cells in five independent multicenter cohorts. Coupled with the markedly enhanced interactions among TAMCs, iCAFs, and T cells via immunosuppressive signaling pathways, including IL‐1 following AG neoadjuvant therapy, we proposed that despite an increase in beneficial immune subsets such as cytotoxic T cells post‐therapy, the co‐existence of TAMCs and iCAFs might impede their functioning, thereby sustaining an immunosuppressive TME and limiting therapeutic efficacy.

MIF, as a pleiotropic cytokine, activates the host's innate and adaptive immunity during pathogenic infection; also, it can accelerate tumor invasion and metastasis by recruiting suppressive Tregs and shaping an immunosuppressive TME.^[^ ^28^, ^29^, ^30^ ^]^ Therapeutic agents targeting MIF, including the small‐molecule inhibitor 4‐IPP and the monoclonal antibody imalumab, have demonstrated promising antitumor activity in recurrent ovarian cancer and metastatic colorectal cancer.^[^ ^38^ ^]^ Furthermore, the MIF antagonist ISO‐1 has already been found to inhibit lung cancer growth and protect adipose tissue from cachexia by targeting the MIF‐ACKR3 axis.^[^ ^39^ ^]^ Recent studies have indicated that targeting the MIF‐mediated immunosuppressive network can convert immunologically “cold” tumors into “hot” tumors, thereby enhancing the efficacy of immunotherapy in breast and colorectal cancers.^[^ ^40^ ^]^ We observed that TAMC ablation, combined with ISO‐1 disruption of MIF‐mediated crosstalk among TAMCs, iCAFs, and T cells induced by AG, could effectively reprogram TME and improve AG efficacy in genetically engineered mouse models. Moreover, the TAMC stabilizer SCG could substitute TAMC ablation in our study, as an effective agent extensively validated in preclinical trials for reducing PDAC growth. When combined with the MIF antagonist ISO‐1, SCG effectively enhanced antitumor immunity and sensitized tumors to AG therapy.

Given that AG neoadjuvant therapy substantially enhanced immunosuppressive crosstalk among TAMCs, iCAFs, and T cells via MIF‐mediated signaling, we sought to evaluate the therapeutic potential of disrupting this axis. Notably, both genetic ablation of TAMCs or their pharmacological stabilization with SCG effectively alleviated this immunosuppressive niche, underscoring TAMCs as a critical cellular target for therapeutic intervention. Concurrently, AG treatment increased the infiltration of effector immune subsets such as CD8_IFN and NKT cells, while reducing the immunosuppressive populations including CD8_Tex, Tregs, and CD4_Tex, thereby establishing an immunologically favorable microenvironment for combination therapy. Importantly, our orthotopic PDAC model demonstrated that inhibition of MIF signaling with ISO‐1 markedly enhanced the efficacy of anti‐PD‐1 therapy in the setting of AG treatment. These results collectively suggest that MIF blockade may overcome resistance to immunotherapy and sensitize PDAC to immune checkpoint inhibition when administered alongside AG‐based neoadjuvant regimens, providing a compelling rationale for further clinical investigation.

Our study provided novel insights into the heterogeneous TMEs shaped by the two first‐line neoadjuvant therapy paradigms for PDAC, and informed the development of subsequent combined therapeutic strategies. Specifically, we highlighted that AG treatment induced TAMC activation, which in turn promoted the transformation of myCAFs into iCAFs and reprogrammed the TME toward an immunosuppressive state. Targeting AG‐induced TAMC activation, together with the enhanced MIF‐mediated crosstalk among TAMCs, iCAFs, and T cells, reactivated antitumor immunity and sensitized tumors to AG. However, this study also had several limitations. First, the multicenter scRNA‐seq datasets integrated in this analysis were generated across varying platforms and over extended time frames. Despite rigorous quality control and batch‐effect correction, some residual variability might remain unavoidable. Second, due to the substantial challenges of acquiring matched pre‐ and post‐neoadjuvant therapy specimens—particularly when relying on fine‐needle aspiration before treatment and surgical resection afterward for scRNA‐seq—our study adopted an unpaired design, comparing treatment‐naive samples with those obtained following various neoadjuvant chemotherapy regimens. While this design has yielded important insights into therapy‐induced alterations within the TME, future investigations incorporating larger cohorts with longitudinally paired clinical specimens will be critical to further validate and expand upon our findings. Third, the efficacy of dual targeting of TAMCs and MIF signaling in enhancing sensitivity to AG therapy and immunotherapy was validated in genetically engineered mouse models and preclinical systems; however, further investigation and validation are warranted to support its translational application.

In conclusion, our study highlighted the dual nature of AG neoadjuvant therapy in PDAC by integrating large‐scale multicenter scRNA‐seq and bulk cohorts. On the one hand, AG intervention promoted the phenotypic shift of residual malignant cells toward a more indolent classical subtype. On the other hand, it also fostered an immunosuppressive niche via TAMC activation, myCAFs‐to‐iCAFs transformation, and enhanced MIF‐mediated crosstalk among these populations. Notably, targeting this immunosuppressive niche with a combination of mast cell stabilizer SCG and MIF antagonist ISO‐1 not only reversed this TME but also enhanced AG chemosensitivity. This effect was further amplified when combined with anti‐PD‐1 therapy, presenting a promising strategy for improving neoadjuvant treatment efficacy in PDAC.

## 4 Experimental Section

### Data Collection and Integration

The official addresses and details of the eight public scRNA‐seq cohorts are summarized in Table S1 (Supporting Information). Specifically, the datasets GSE154778,^[^ ^41^ ^]^ GSE155698,^[^ ^42^ ^]^ GSE156405,^[^ ^17^ ^]^ GSE202051,^[^ ^18^ ^]^ GSE205013,^[^ ^43^ ^]^ GSE212966,^[^ ^44^ ^]^ and GSE217845^[^ ^45^ ^]^ were retrieved from the Gene Expression Omnibus (GEO, http://www.ncbi.nlm.nih.gov/geo/) database. The CRA001160^[^ ^46^ ^]^ dataset was downloaded from Genome Sequence Archive (GSA, https://ngdc.cncb.ac.cn/gsa/) website. Moreover, two spatial transcriptome datasets, GSE235315^[^ ^47^ ^]^ and GSE272362,^[^ ^48^ ^]^ were also downloaded from the GEO portal (Table S2, Supporting Information).

After quality control, the dimensionality reduction and cell subtype identification were achieved using the standard pipeline of the Seurat package, which included the following steps: 1) The NormalizeData function was used to normalize the eligible cells, and the FindVariableFeatures function was employed to identify the top 3000 most variable genes. 2) Principal component analysis was performed on the datasets after regression using unique molecular identifiers, cell cycle scores, percentage of mitochondrial genes, cohorts, and samples. 3) The Harmony package^[^ ^49^ ^]^ was used to remove batch effects from the eight cohorts and samples. 4) The FindNeighbors function was used to determine the nearest neighbors for graph clusters according to the top 30 principal components, and the FindClusters function was used to obtain the final clusters. 5) The FindAllMarkers function was used to conduct differential expression analysis of each cluster. Then, cell subtypes were annotated by referring to the previous authoritative literature and the results of benign and malignant inference by InferCNV package.^[^ ^50^ ^]^

The prognostic significance and the molecular characteristics of specific cell subtypes in large‐scale multicenter cohorts with comprehensive clinical annotations were validated by acquiring and integrating multi‐institutional datasets with the following approach and transforming them into the log2 (TPM+1) format for subsequent analysis:

***TCGA & GTEx***: The TCGA‐PDAC and GTEx‐Pancreas datasets were downloaded from the UCSC Xena (http://xena.ucsc.edu/) website.

***ArrayExpress***: The E‐MTAB‐6134 dataset was retrieved from the ArrayExpress portal (https://www.ebi.ac.uk/biostudies/arrayexpress/).

***ICGC***: The PDAC_AU_array, PDAC_AU_seq and PDAC_CA_seq cohorts were obtained from the International Cancer Genome Consortium (ICGC, https://dcc.icgc.org/).

***GEO***: Finally, the datasets GSE28735, GSE62452, and GSE71729 with tumor and adjacent normal tissues were curated from the GEO database. The basic information of these datasets is shown in Table S3 (Supporting Information).

### Cell–Cell Communication

The effect of neoadjuvant therapy on the interaction among various components within the TME was explored using the CellChat package^[^ ^51^ ^]^ to infer the cell–cell communication network.

### Cell Proportion Analysis

To evaluate the distribution preferences of cell subsets across different groups, two well‐established methods were employed: the ratio of observed to expected (Ro/e) index^[^ ^52^ ^]^ and the odds ratio (OR) value.^[^ ^53^ ^]^ The Ro/e (observed/expected) index was calculated to quantify tissue‐specific enrichment or depletion of cell subpopulations in specific groups. The expected cell number for each cell subset and group were derived from the chi‐squared test. A Ro/e index > 1 indicates enrichment of the cell subtype in that tissue, while a value < 1 indicates depletion. In addition, to calculate the OR value, 2 × 2 contingency tables were constructed for each cell subset as well as group. Then, Fisher's exact test was used to compute OR values and raw *P* values, which were further adjusted using the Benjamini‐Hochberg (BH) method. An OR > 1.5 with an adjusted *P* value < 1×10^−10^ was interpreted as a significant preferential distribution in a given tissue, while an OR < 0.5 indicated a significant avoidance.

### Functional Enrichment Analysis

Gene Ontology (GO), Kyoto Encyclopedia of Genes and Genomes (KEGG), and gene set enrichment analysis (GSEA) were conducted using the signature markers of each cell subset to extract the distinct biological characteristics. The enriched pathways with adjusted *P* values below 0.05 and top normalized enrichment scores were considered significantly enriched.

### Gene Set Scores for Single‐Cell and Bulk Data

To evaluate gene expression signatures in single cells, the AUCell, UCell, singscore, ssGSEA, JASMINE, and viper function in the irGSEA package,^[^ ^54^ ^]^ as well as the AddModuleScore function of Seurat package, were used to score the expression level of several common TAMC activation gene sets (*KIT*, *FCER1G*, *CPA3*, *TPSAB1*, and *TPSB2*); their sum was used as the TAMC activation score. Bisque,^[^ ^12^ ^]^ a robust deconvolution algorithm, was used to evaluate the proportion of cellular components from scRNA‐seq data in bulk samples of transcriptomic cohorts.

### Determining Malignant Cell Lineage States

To characterize heterogeneous malignant cell lineages, established markers were utilized for classical and basal PDAC subtypes from previous studies (Table S4, Supporting Information).^[^ ^19^, ^22^, ^23^ ^]^ In accordance with the methodology described by Pei et al.,^[^ ^22^ ^]^ the AddModuleScore function was employed in the Seurat package to calculate module score for classical and basal signature gene sets within the scRNA‐seq data. A cell was classified as classical if its classical module score exceeded the basal module score by a threshold of > 0.3. Conversely, a cell was assigned to the basal state if its basal module score surpassed the classical score by > 0.3. Cells not meeting either criterion were categorized as intermediate.

### Cell Lines and Co‐Culture Experiments

The human PDAC cell line PANC‐1 (*RRID*: CVCL_0480) was obtained from the American Type Culture Collection (*ATCC*, Manassas, USA). The human mast cell line LAD‐2 (*RRID*: CVCL_0387) and the murine mast cell line MC/9 (*RRID*: CVCL_0408) were purchased from the Shanghai Cell Bank of the BLUEFBIO (Shanghai, China). The murine PDAC cell line from KPC (LSL‐Kras^G12D/+^; LSL‐Trp53^R172H/+^; Pdx‐1‐Cre) mouse was a gift from Dr. Tingbo Liang (The First Affiliated Hospital, Zhejiang University School of Medicine, Hangzhou, China). The PANC‐1 was cultured in DMEM containing 10% FBS in an incubator at 37 °C and in the presence of 5% CO_2_. The LAD‐2 was cultured in StemPro‐34 serum‐free media containing 100 U mL^−1^ penicillin, 100 µg mL^−1^ streptomycin, and 100 ng mL^−1^ recombinant human stem cell factor.^[^ ^24^ ^]^ For co‐culture experiments, PANC‐1 cells were cultured for 24 h in a blank control, AG regimen containing 500 nM gemcitabine and 50 nM nab‐paclitaxel, and FOLFIRINOX regimen containing 4 µM fluorouracil, 4 µM oxaliplatin, and 2 µM irinotecan.^[^ ^55^, ^56^ ^]^ The supernatants of PANC‐1 cells from the aforementioned three groups were filtered and mixed with LAD‐2 medium in a ratio of 1:1, and LAD‐2 cells were continuously cultured for 48 h for subsequent detection. Furthermore, the effect of activated TAMCs on CAFs was investigated by culturing isolated primary CAFs from fresh PDAC tissues in a ratio of 1:1 of DMEM complete medium and LAD‐2 culture supernatant stimulated by the aforementioned three interventions. This was followed by detection and analysis.

Further, the murine KPC cell line was cultured using RPMI‐1640 medium containing 10% FBS and stimulated it with blank control, AG regimen, and FOLFIRINOX regimen for 24 h. Subsequently, the aforementioned filtered supernatant was co‐cultured with the dedicated medium [RPMI‐1640 containing 10% FBS supplemented successively with 4 mM L‐glutamine (Gibco), 50 µM β‐mercaptoethanol (Sigma), 1 mM sodium pyruvate (Gibco), 100 µM nonessential amino acids (Gibco), 100 U mL^−1^ penicillin, 100 µg mL^−1^ streptomycin, 25 mM HEPES (Gibco), and 30 ng mL^−1^ murine IL‐3 (Peprotech)] in a ratio of 1:1 for murine mast cell line MC/9, followed by subsequent tests. All cell lines were confirmed mycoplasma‐free using the MycoAlert Mycoplasma Detection Kit (Lonza) prior to experiments.

### Western Blot Experiments

Equal amounts of protein (20 µg per lane), quantified using a BCA Protein Quantification Kit (Vazyme, Cat#: E112‐01, Nanjing, China), were separated on 7.5‐12.5% gradient SDS‐PAGE gels and subsequently transferred to PVDF membranes (Immobilon‐P, 0.45 µm, Cat#: IPVH00010, Millipore, USA). The membranes were blocked with 5% non‐fat milk in TBST buffer for 2 h at room temperature and then incubated overnight at 4 °C with the following primary antibodies: anti‐FcεRI (Cat#: NBP1‐43279, 1:1000; Novus), anti‐c‐Kit (Cat#: ab283653, 1:1000; Abcam), anti‐c‐Kit (Cat#: ab273119, 1: 1000; Abcam), anti‐α‐SMA (Cat#: ab7817, 1:1000; Abcam), and anti‐IL‐6 (Cat#: ab233706, 1:1000; Abcam). After washing with TBST, the membranes were incubated for 2 h at room temperature with HRP‐conjugated secondary antibodies: anti‐rabbit IgG‐HRP(Cat#: 7074, 1:5000, Cell Signaling Technology) or anti‐mouse IgG‐HRP (Cat#: 7076, 1:5000, Cell Signaling Technology). Following a final wash, immunoreactive bands were visualized using Immobilon Western Chemiluminescent HRP Substrate (Cat#: WBKLS0500, Millipore, USA) and captured on a Tanon Chemiluminescent Imaging System (Tanon, Shanghai, China).

### Flow Cytometry

The activation status of LAD‐2 cells was determined by flow cytometry using the following antibodies: anti‐human CD117 (c‐kit, Cat#: 313204, 0.2 mg mL^−1^; BioLegend) and anti‐human FcεR1α (Cat#: 747787, 0.2 mg mL^−1^; BD Biosciences). The activation status of MC/9 cells was determined using the following antibodies: anti‐mouse CD117 (c‐kit, Cat#: 155108, 0.2 mg mL^−1^; BioLegend) and anti‐mouse FcεR1α (Cat#: 750852, 0.2 mg mL^−1^; BD Biosciences). In addition, flow cytometry was performed with anti‐human IL‐6 (Cat#: 501106, 0.2 mg mL^−1^; BioLegend) and anti‐α‐SMA (Cat#: ab7817, 1.137 µg mL^−1^; Abcam) conjugated fluorescent secondary antibodies (Alexa Fluor 488, Cat#: A‐10680, 1:2000; Invitrogen) to evaluate the effect of activated TAMCs on CAFs.

Furthermore, T‐cell exhaustion levels were evaluated in both subcutaneous xenograft and orthotopic tumor models by targeting two hallmark immune checkpoint molecules using the following antibodies: anti‐mouse PD‐1 (Cat#: 562584, 0.2 mg mL^−1^; BD Biosciences) and anti‐mouse TIM‐3 (Cat#: 568796, 0.2 mg mL^−1^; BD Biosciences). On the contrary, the proportions of tumor‐infiltrating effector T cells in the microenvironment were also determined using a standardized antibody panel including anti‐mouse IFN‐γ (Cat#: 563376, 0.2 mg mL^−1^; BD Biosciences), anti‐mouse TNF‐α (Cat#: 554419, 0.2 mg mL^−1^; BD Biosciences), and anti‐mouse Granzyme B (Cat#: 25‐8898‐82, 0.2 mg mL^−1^; Invitrogen).

### Animal Models

C57BL/6 and B6 Albino mice were obtained from Weitong Lihua Biotechnology (Beijing, China), while B6.Cg‐Kit^W‐sh^/HNihrJaeBsmGlliJ [Kit^W‐sh^] mice were obtained from Jackson Laboratories (ME, USA). All mice were randomly assigned to different groups after 1 week of adaptive feeding under standard conditions. The orthotopic tumor model was established by orthotopically injecting 5 × 10^4^ luciferase‐expressing murine KPC cells into the pancreas of each mouse and monitoring their growth using a small‐animal in vivo imaging system (IVIS Spectrum, PerkinElmer, Waltham, MA, USA). The subcutaneous tumor model was established by subcutaneously injecting 1.5 × 10^6^ KPC cells into the groin of each mouse. The tumor growth was monitored using a vernier caliper, and the volume was calculated using the following formula: volume = 1/2*ab*,^2^ where *a* is the long axis and *b* is the short axis. All animal procedures in this study were approved by the ethics committee of the Tianjin Medical University Cancer Institute and Hospital (Approval No. NSFC‐AE‐2025373).

### Immunohistochemistry and TUNEL Staining

Mouse tumor tissues were harvested, fixed in formalin, embedded in paraffin, and sectioned for histological analysis. For immunohistochemical (IHC) staining, tissue sections were incubated with a primary anti‐cleaved caspase‐3 antibody (Cat#: 9664, 1:2000; Cell Signaling Technology), followed by signal detection using the M&R HRP/DAB Detection IHC Kit (Vazyme, Cat#: HC301‐01, Nanjing, China). Additionally, TUNEL staining was performed using the In situ Cell Death Detection Kit (POD; Roche, Basel, Switzerland) according to the manufacturer's instructions. The percentages of positively stained areas were quantified using Image J software.

### Statistical Analysis

The data processing, analysis, and visualization for this study were performed using R 4.4.3. The survminer package was used to determine the optimal cutoff value. Kaplan‐Meier curves were used for survival analysis. The Student's *t* test or Wilcoxon rank‐sum test was conducted for comparing continuous variables between the two groups. Two‐sided *P* values less than 0.05 indicated statistically significant differences. Flow cytometry data were derived from three independent replicates. Statistical significance was assessed using the Student's *t* test. The error bars in the figures represent the standard deviation (SD).

advs72205‐sup‐0002‐Supplementary table.xlsx

## Conflict of Interest

The authors declare no conflict of interest.

## Author Contributions

L.W. and G.S. contributed equally as co‐first authors in this article. L.W., G.S., G.X., Y.X., and T.Z. performed conceptualization, data curation, and wrote the draft, formal analysis, and methodology. Z.K.L., X.M., M.L., Z.Y.L., Y.W., Z.J.L., Q.F., H.S., N.Z., and C.Y. performed formal analysis and methodology. J.H. performed funding acquisition. J.H., J.Y., Y.X., and T.Z. performed supervision and reviewed the draft. All authors have read and approved the article.

## Supporting information



Supporting Information

Supporting Table

## Data Availability

The eight scRNA‐seq, two spatial transcriptomes, and nine bulk transcriptome cohorts included in this study are publicly available through the web addresses summarized in Tables S1–S3. This study did not generate original codes. All software and algorithms used in this study are publicly available and listed in the corresponding methods section. Any additional information required to reanalyze the data reported in this paper is available from the corresponding author upon request.
