# Supplementary material for: Targeting Mast Cell Activation and MIF‐Mediated Remodelling Enhances Chemotherapy Response in Pancreatic Cancer
Source: Adv Sci (Weinh). 2025 Oct 29;12(48):e09930. doi: 10.1002/advs.202509930 (PMC12752552; doi:10.1002/advs.202509930)
Supplement: Supplementary file 1 — Supporting Information [file ADVS-12-e09930-s002.docx]

**Supplementary figures for:**

Targeting Mast Cell Activation and MIF-mediated Remodelling Enhances Chemotherapy Response in Pancreatic Cancer

Libo Wang^#1^, Guangcong Shen^#1^, Guanpeng Xie^1^, Zekun Li^1^, Xiaoqing Ma^1^, Mengyu Li^1^, Ziyun Liu^1^, Yadi Wang^1^, Zongjing Lv^1^, Qingxiao Fang^1^, Huihui Sun^1^, Ningning Zhao^1^, Chao Yang^1^, Tianxing Zhou^2^*, Yongjie Xie^3^*, Jun Yu^4^*, Jihui Hao^5^*

***Correspondence**: Pancreas Center, Tianjin Medical University Cancer Institute and Hospital, National Clinical Research Center for Cancer, Tianjin, People's Republic of China. Electronic address: [haojihui@tjmuch.com](mailto:haojihui@tjmuch.com).

**Supplementary figures**

- Figure S1. Data quality control and cell annotation.
- Figure S2. UMAP plots of classical markers for each major cell subpopulation.
- Figure S3. Distribution preference of 13 major cell types across several common clinical features.
- Figure S4. Activation level of TAMC in each cluster.
- Figure S5. Cell-cell communication network among TAMCs and 13 major cell subsets.
- Figure S6. Prognostic value of TAMCs and activated TAMCs.
- Figure S7. Functional enrichment analysis uncovers specific biological pathways among different CAF subtypes.
- Figure S8. Cell-cell communication between TAMCs and six CAF subtypes.
- Figure S9. Evolution of malignant cell lineages by different treatment modalities.
- Figure S10. Cell-cell communication network among TAMCs, tumor cells, and six CAF subtypes.
- Figure S11. Integrated analysis of single-cell RNA sequencing and spatial transcriptome as well as bulk transcriptome data.
- Figure S12. Cell-cell communication between TAMCs, six CAF subtypes, and 10 T-cell subtypes.
- Figure S13. Experimental validation of targeting TAMCs and MIF signaling to reverse immunosuppressive microenvironment.


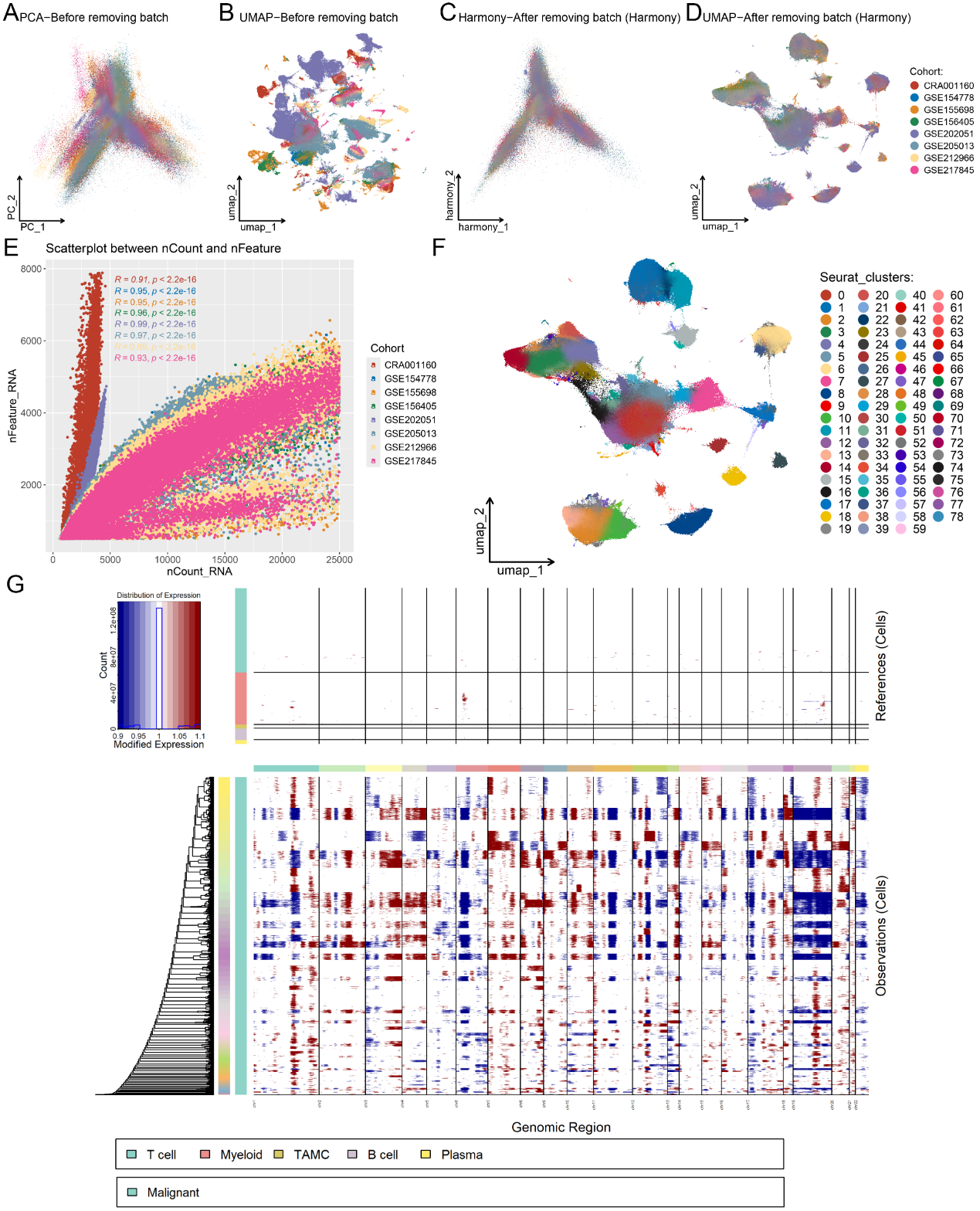


**Figure S1.** Data quality control and cell annotation. A) The principal component analysis (PCA) plot before removing batch effect. B) The uniform manifold approximation and projection (UMAP) plot before removing batch effect. C) The PCA plot after removing batch effect using Harmony algorithm. D) The UMAP plot after removing batch effect using Harmony algorithm. E) The correlation of the total number of UMIs (nCount_RNA) with the detected genes (nFeature_RNA) in eight cohorts. F) The UMAP plot for 79 clusters identified by unsupervised clustering. G) The distribution of inferred copy number variation (inferCNV) on chromosomes.


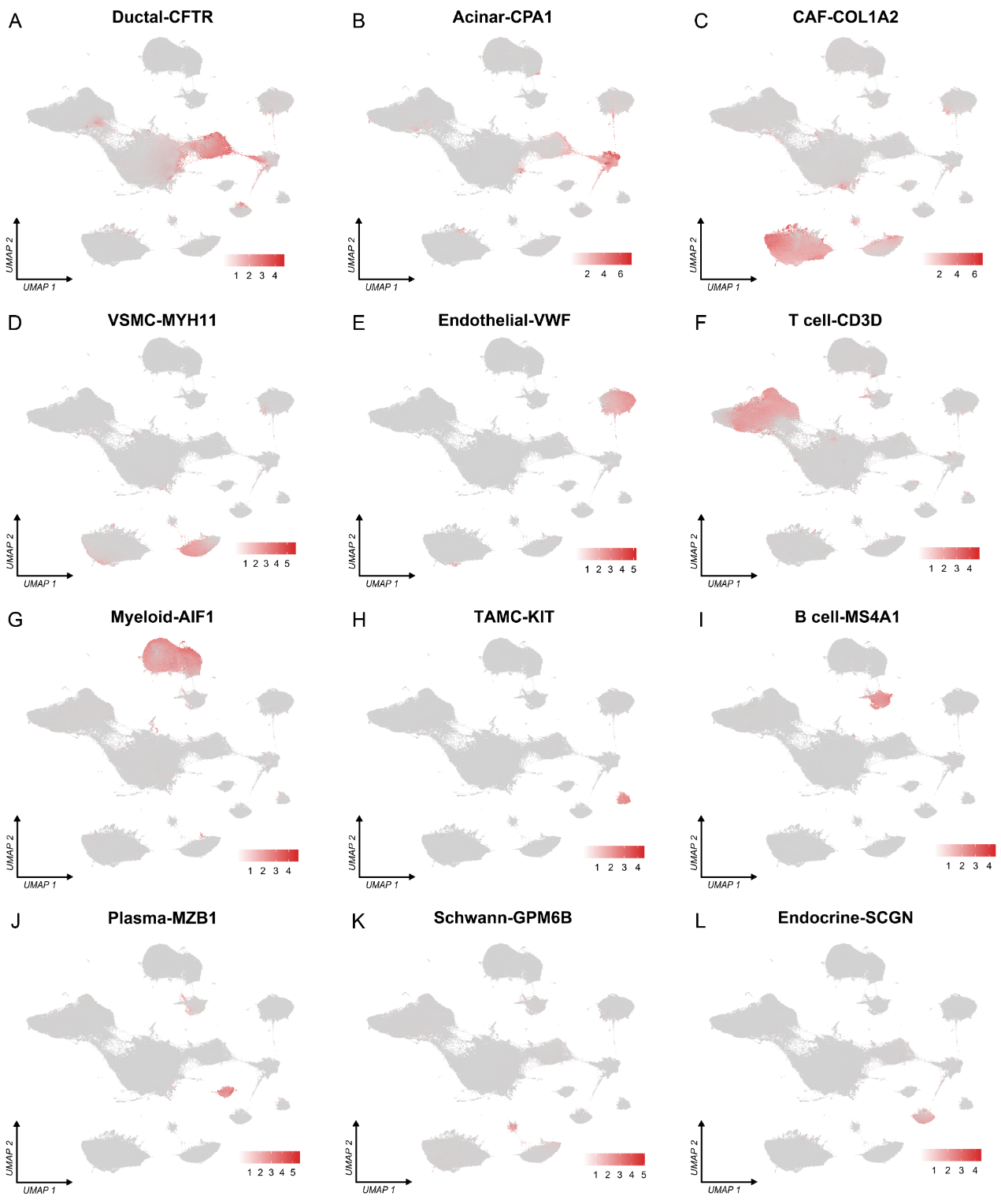


**Figure S2.** UMAP plots of classical markers for each major cell subpopulation. A-L) UMAP plots showing the expression of the classical ductal cell marker *CFTR* (A), acinar cell marker *CPA1* (B), cancer-associated fibroblast (CAF) marker *COL1A2* (C), vascular smooth muscle cell (VSMC) marker *MYH11* (D), endothelial cell marker *VWF* (E), T cell marker *CD3D* (F), myeloid cell marker *AIF1* (G), tumor-associated mast cell (TAMC) marker *KIT* (H), B cell marker *MS4A1* (I), plasma cell marker *MZB1* (J), schwann cell marker *GPM6B* (K), and endocrine cell marker *SCGN* (L).


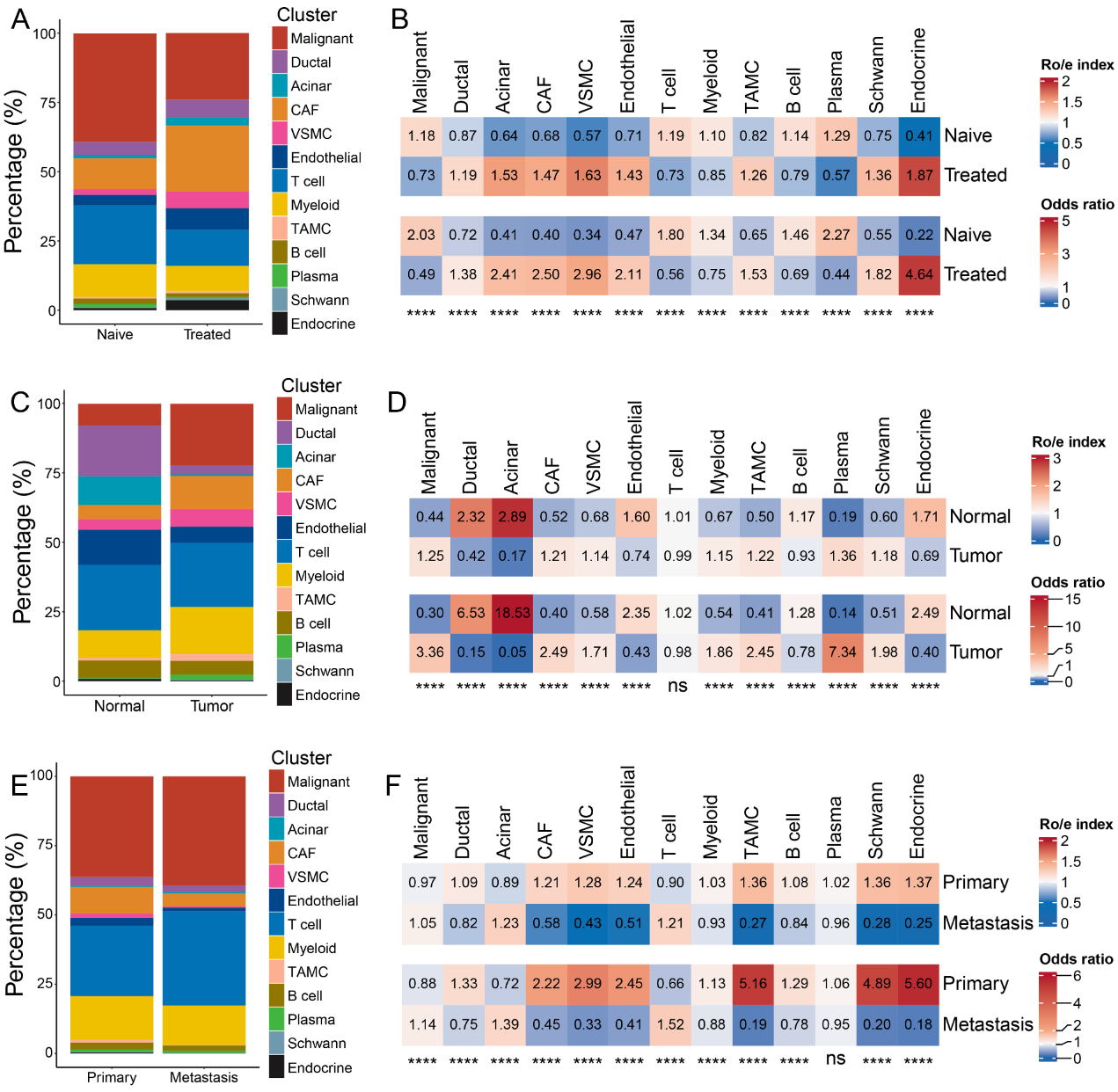


**Figure S3.** Distribution preference of 13 major cell types across several common clinical features. A) The percentage of 13 major cell types in naive and treated groups. B) Ro/e index and odds ratio (OR) value reflecting the distribution preferences of these cell types between the naive and treated groups. C) The percentage of 13 major cell types in normal and tumor tissues. D) Ro/e index and OR value reflecting enrichment or depletion of these cell types in normal and tumor tissues. E) The percentage of 13 major cell types in primary and metastatic tumor samples. F) Ro/e index and OR value demonstrating distribution preferences of cell types in primary and metastatic sites. Ro/e denotes the ratio of observed to expected cell number; OR indicates the odds ratio for tissue-distribution preferences. A Ro/e index > 1 or OR-value > 1.5 suggests enrichment of the cell subtype in the tissue, while Ro/e index < 1 or OR-value < 0.5 indicates depletion. **P* < 0.05, ***P* < 0.01, ****P* < 0.001, *****P* < 0.0001.


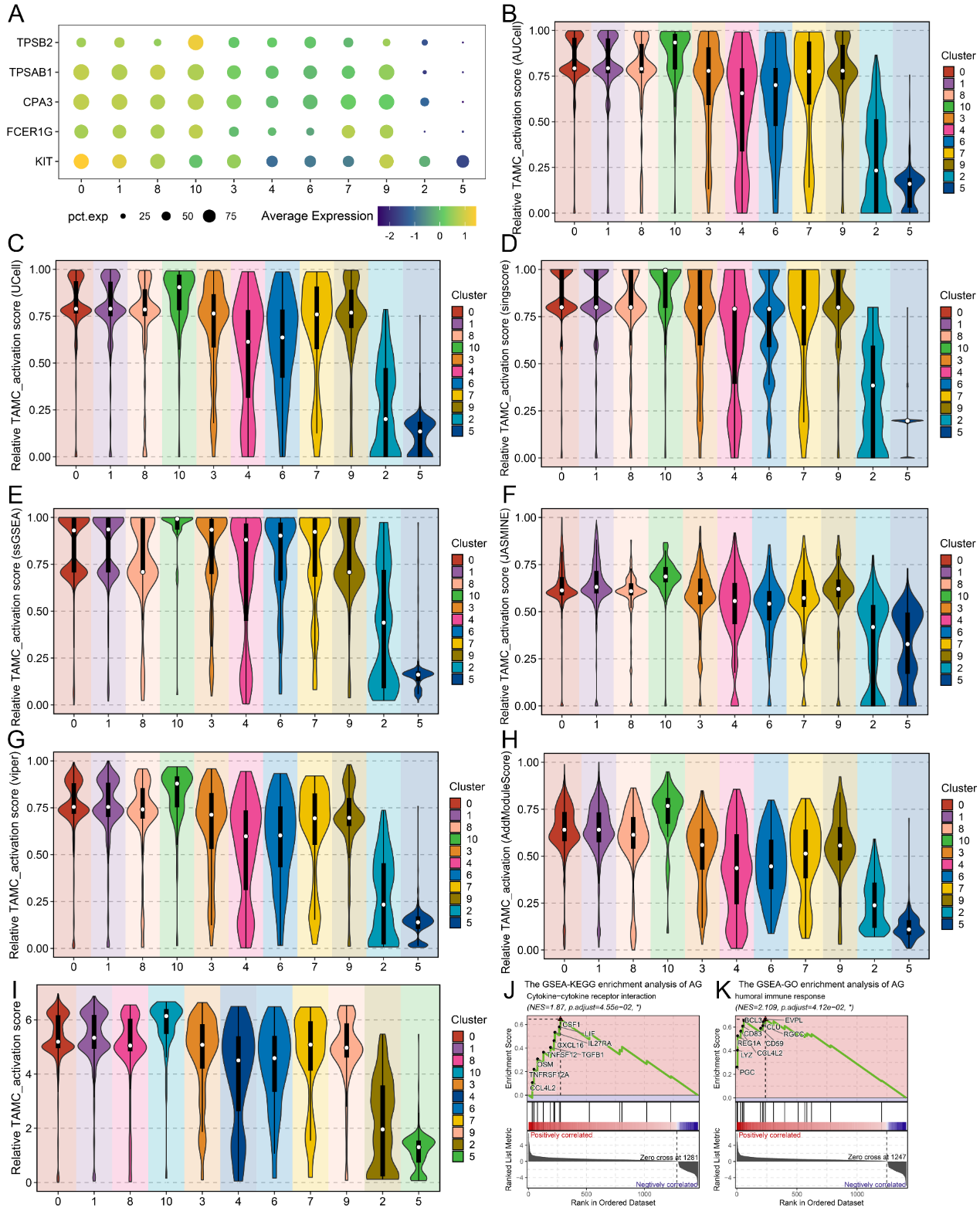


**Figure S4.** Activation level of TAMC in each cluster. A) Dot plots exhibiting the expression levels of 5 hallmark activation markers in 11 tumor-associated mast cell (TAMC) clusters resulting from unsupervised clustering. B-I) Violin plot showing TAMC activation levels derived from AUCell (B), UCell (C), singscore (D), ssGSEA (E), JASMINE (F), viper (G), AddModuleScore (H), and the total activation scores (I) accumulated by seven algorithms in 11 clusters. J, K) Gene set enrichment analysis highlights the significant biological pathways in mast cells of the AG group using Kyoto Encyclopedia of Genes and Genomes (KEGG) and Gene Ontology (GO) databases.
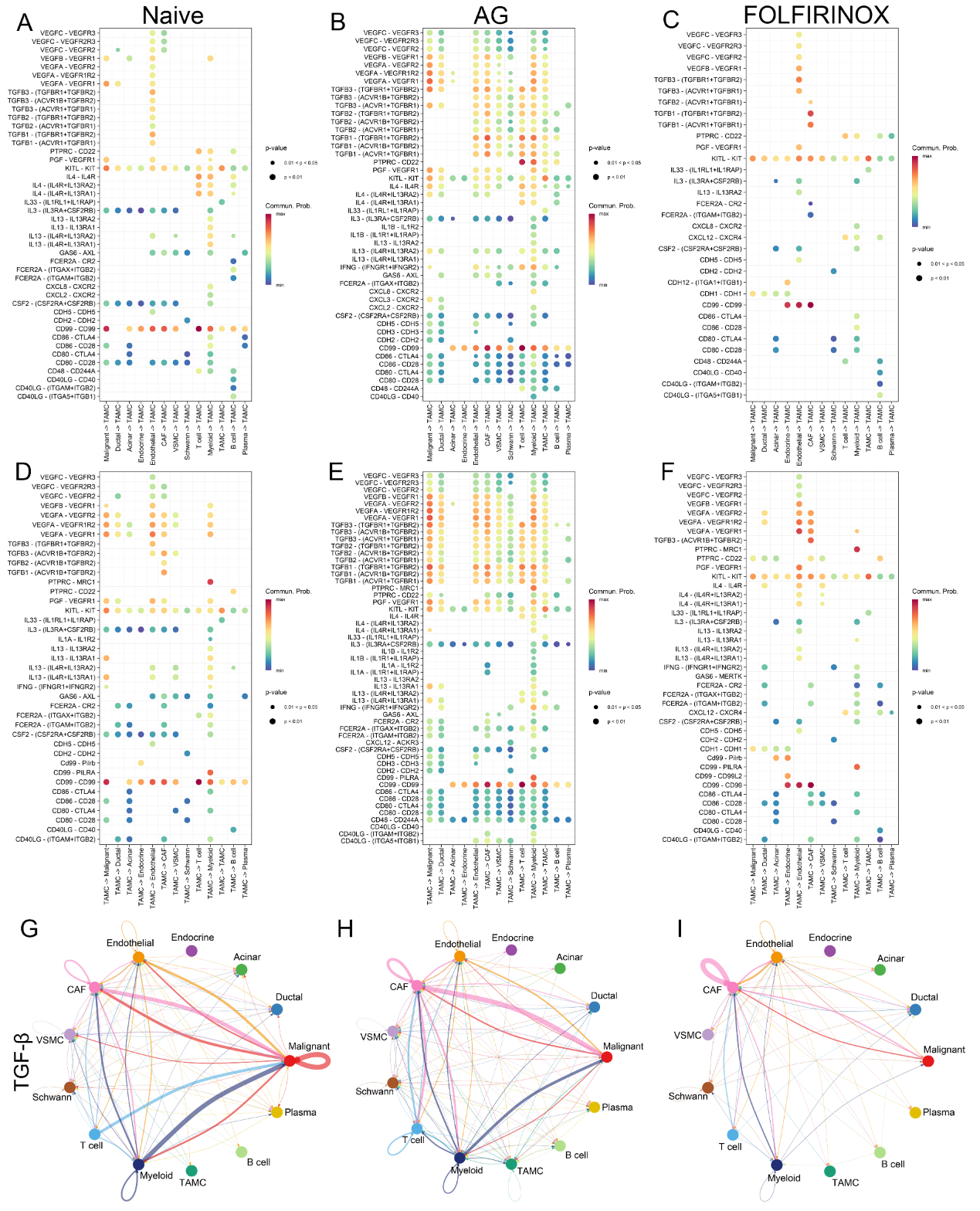


**Figure S5.** Cell-cell communication network among TAMCs and 13 major cell subsets. A-C) Cell-cell communication networks between 13 major cell subsets and tumor-associated mast cells (TAMCs) in the naive (A), AG (B), and FOLFIRINOX (C) groups. *P* values are indicated by circle size (permutation test). D-F) Cell-cell communication networks between TAMCs and 13 major cell subsets in the naive (D), AG (E), and FOLFIRINOX (F) groups. *P* values are indicated by circle size (permutation test). G-I). Cell-cell communication networks on TGF-β signaling between 13 major cell subsets in the naive (G), AG (H), and FOLFIRINOX (I) groups.


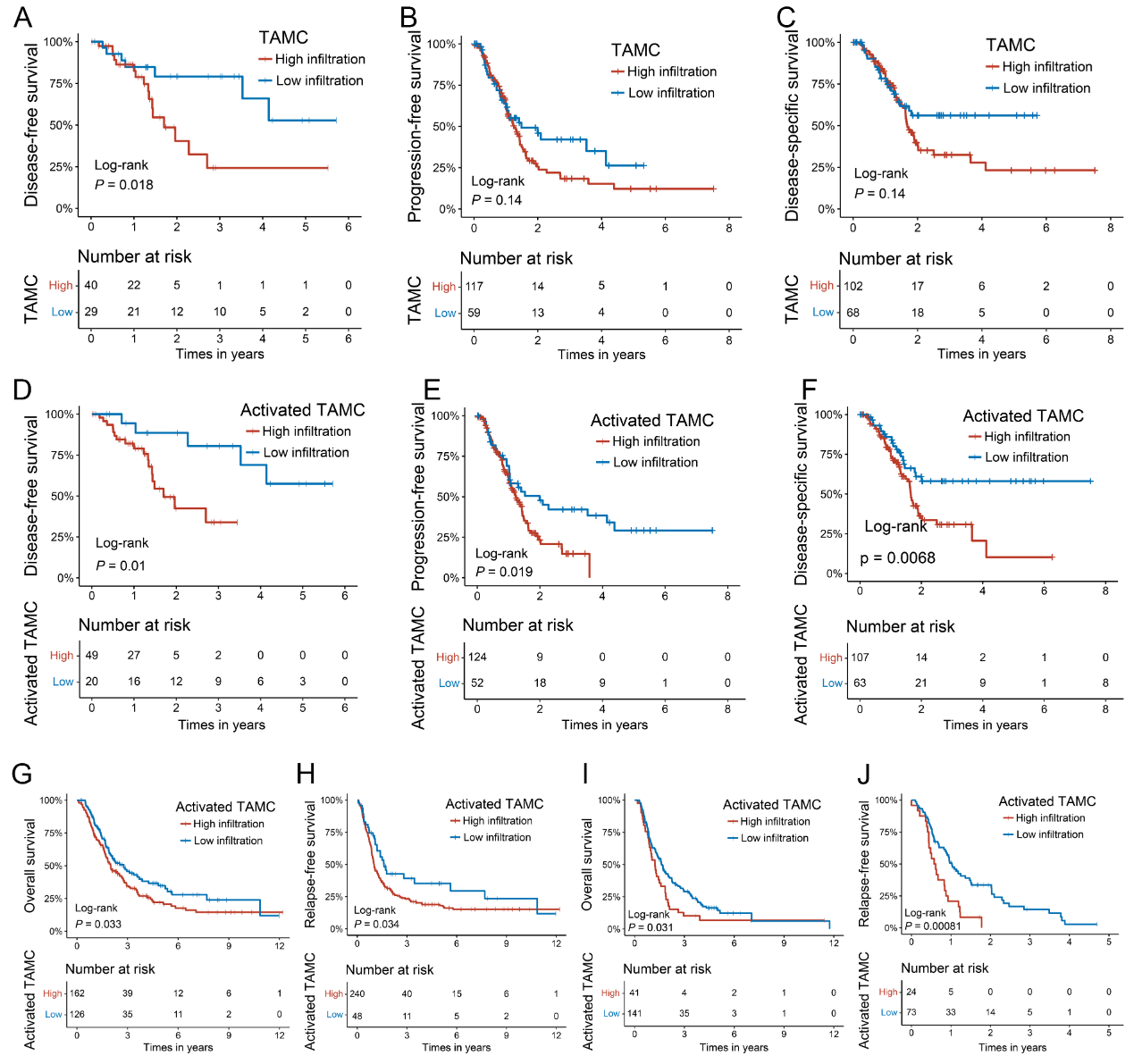


**Figure S6.** Prognostic value of TAMCs and activated TAMCs. A-C) Kaplan-Meier survival curve for disease-free survival (DFS, A), progression-free survival (PFS, B), and disease-specific survival (DSS, C) between groups with high and low estimated proportion of tumor-associated mast cells (TAMCs) in the TCGA-PDAC cohort. D-F) Kaplan-Meier survival curve for DFS (D), PFS (E), and DSS (F) between groups with high and low estimated proportion of activated TAMCs in the TCGA-PDAC cohort. G, H) Kaplan-Meier survival curve for overall survival (OS, G) and relapse-free survival (RFS, H) between groups with high and low estimated proportion of activated TAMCs in the E-MTAB-6134 cohort. I, J) Kaplan-Meier survival curve for OS (I) and RFS (J) between groups with high and low estimated proportion of activated TAMCs in the PDAC_CA_seq cohort. **P* < 0.05, ***P* < 0.01, ****P* < 0.001, *****P* < 0.0001.


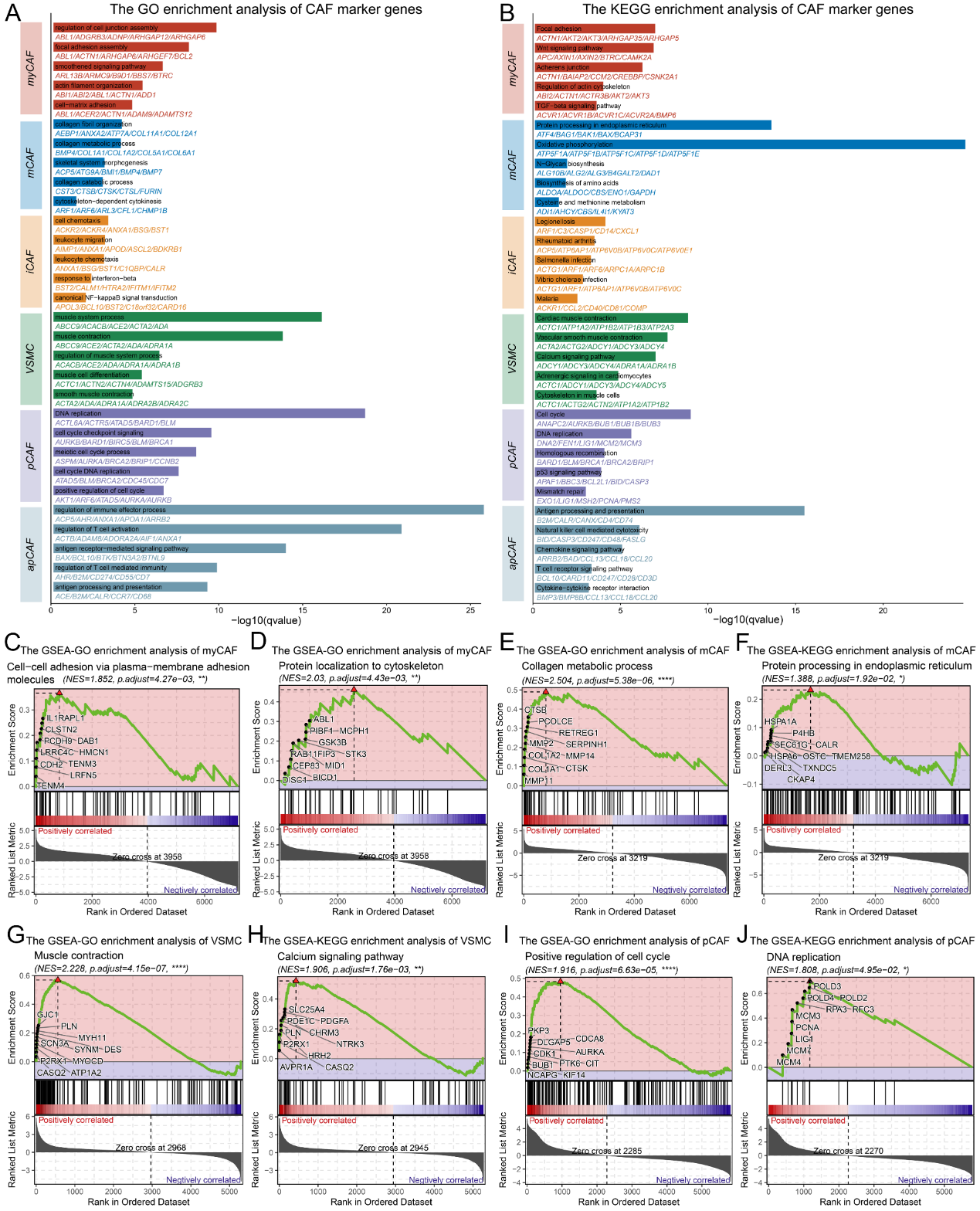


**Figure S7.** Functional enrichment analysis uncovers specific biological pathways among different CAF subtypes. A, B) Gene Ontology (GO, A) and Kyoto Encyclopedia of Genes and Genomes (KEGG, B) functional enrichment analysis of signature genes in six cancer-associated fibroblast (CAF) subtypes. C, D) Gene set enrichment analysis (GSEA) using the GO database highlighted the significant biological pathways of myofibroblastic CAFs. E, F) GSEA analysis using the GO and KEGG databases highlighted the significant biological pathways of matrix CAFs. G, H) GSEA analysis using the GO and KEGG databases highlighted the significant biological pathways of vascular smooth muscle cells (VSMCs). I, J) GSEA analysis using the GO and KEGG databases highlighted the significant biological pathways of proliferative CAFs. **P* < 0.05, ***P* < 0.01, ****P* < 0.001, *****P* < 0.0001.


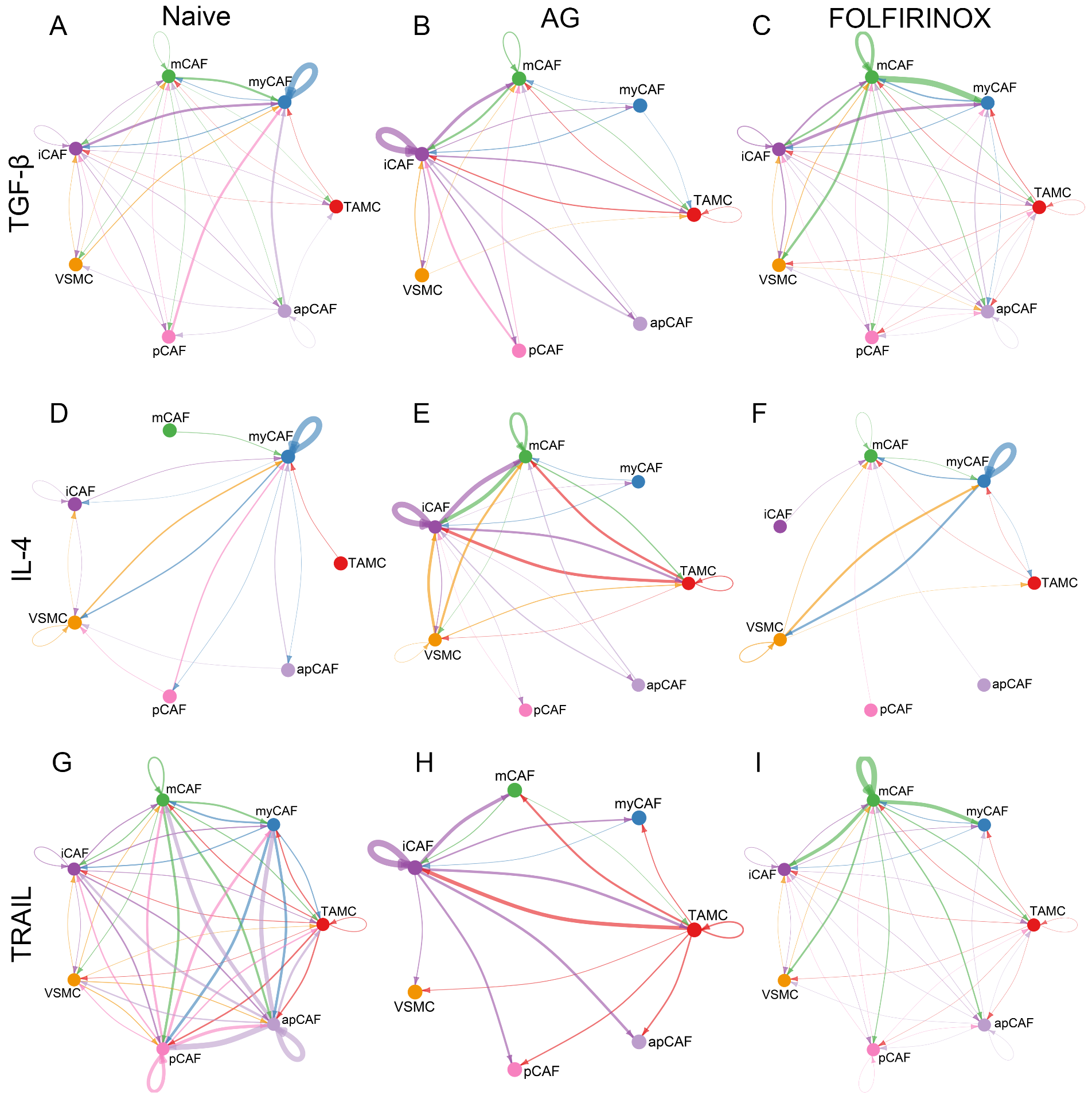


**Figure S8.** Cell-cell communication between TAMCs and six CAF subtypes. A-C) Cell-cell communication networks on TGF-β signaling between tumor-associated mast cells (TAMCs) and six cancer-associated fibroblast (CAF) subtypes in the naive (A), AG (B), and FOLFIRINOX (C) groups. D-F) Cell-cell communication networks on IL-4 signaling between TAMCs and six CAF subtypes in the naive (D), AG (E), and FOLFIRINOX (F) groups. G-I) Cell-cell communication networks on TRAIL signaling between TAMCs and six CAF subtypes in the naive (G), AG (H), and FOLFIRINOX (I) groups.


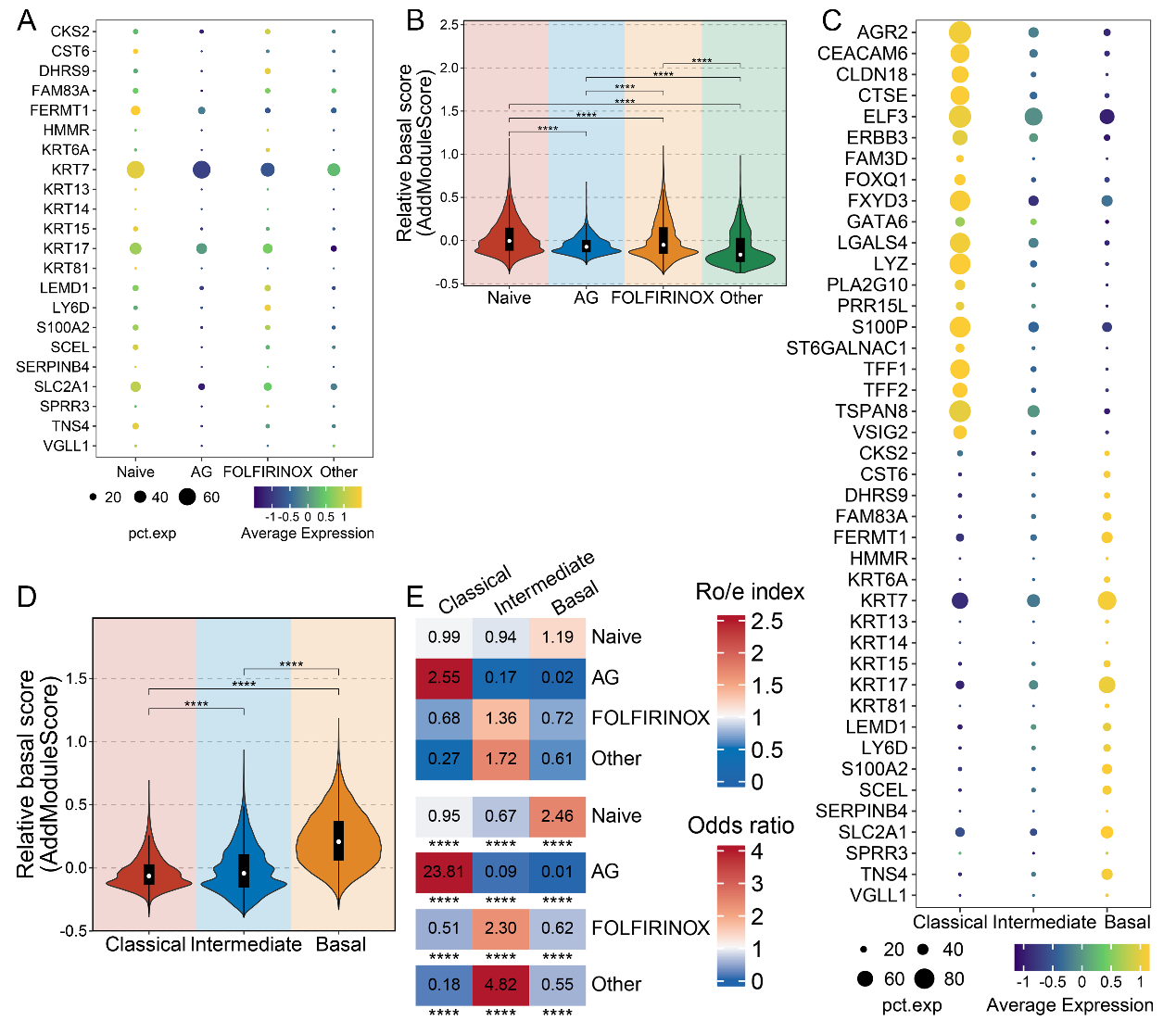


**Figure S9.** Evolution of malignant cell lineages by different treatment modalities. A) Dot plots showing the mean expression levels of basal markers in tumor cells from the four groups of patients. B) Violin plots showing basal signature scores calculated by AddModuleScore function in tumor cells from four groups of patients. C) Dot plots showing the mean expression levels of classical and basal markers in tumor cells from the three malignant cell lineage subtypes. D) Violin plots showing basal signature scores calculated by AddModuleScore function in tumor cells from three malignant cell lineage subtypes. E) Ro/e index and OR value demonstrating distribution preferences of three malignant cell lineage subtypes in the naive group and treatment groups with different treatment regimens. Ro/e denotes the ratio of observed to expected cell number; OR indicates the odds ratio for tissue-distribution preferences. A Ro/e index > 1 or OR-value > 1.5 suggests enrichment of the cell subtype in the tissue, while Ro/e index < 1 or OR-value < 0.5 indicates depletion. **P* < 0.05, ***P* < 0.01, ****P* < 0.001, *****P* < 0.0001.


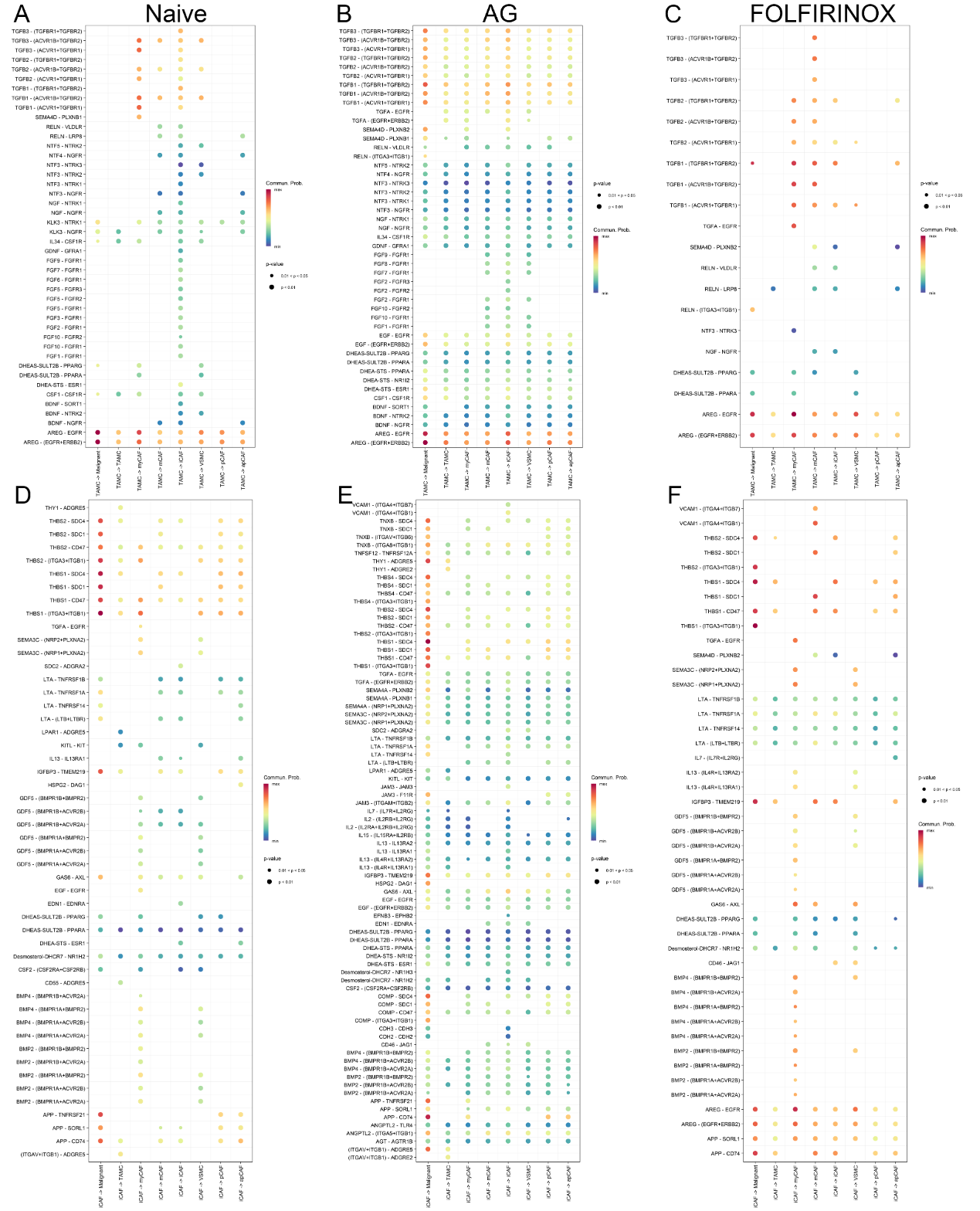


**Figure S10.** Cell-cell communication network among TAMCs, tumor cells, and six CAF subtypes. A-C) Cell-cell communication networks between tumor-associated mast cells (TAMCs) and tumor cells, TAMCs as well as six cancer-associated fibroblast (CAF) subtypes in the naive (A), AG (B), and FOLFIRINOX (C) groups. *P* values are indicated by circle size (permutation test). D-F) Cell-cell communication networks between inflammatory CAFs (iCAFs) and tumor cells, TAMCs as well as six CAF subtypes in the naive (D), AG (E), and FOLFIRINOX (F) groups. *P* values are indicated by circle size (permutation test).


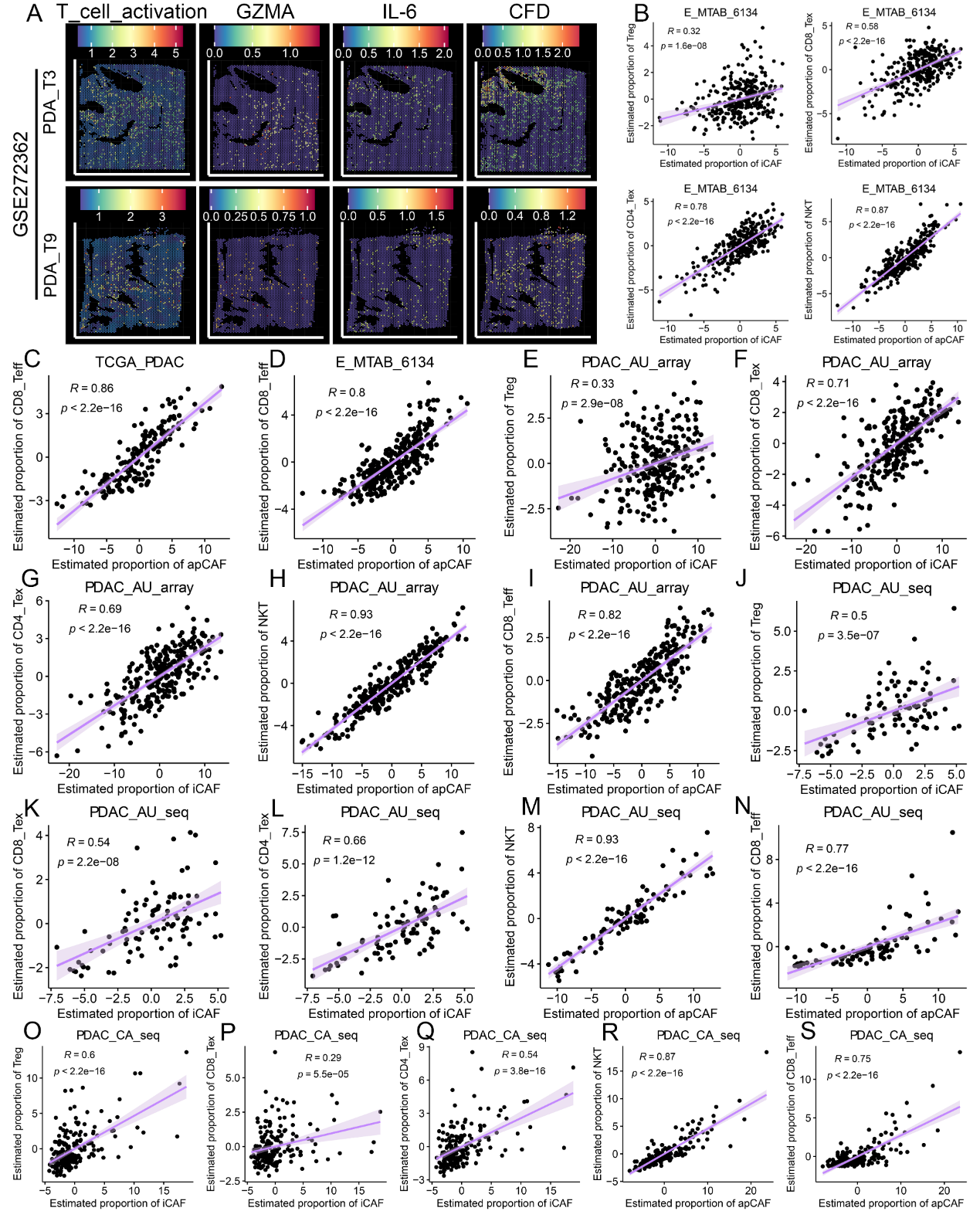


**Figure S11.** Integrated analysis of single-cell RNA sequencing and spatial transcriptome as well as bulk transcriptome data. A) Spatial feature plots of the seven algorithms T-cell activation score, T-cell activation marker GZMA, as well as inflammatory cancer-associated fibroblasts (iCAFs) markers IL-6 and CFD in T3 and T9 tissue sections (from left to right, GSE272362). B) The correlations between the estimated proportions by Bisque deconvolution algorithm of iCAFs and regulatory T cells (Tregs), exhausted CD8^+^ T cells (CD8_Tex), and exhausted CD4^+^ T cells (CD4_Tex), as well as antigen-presenting CAFs (apCAFs) and natural killer T (NKT) cells in the E-MTAB-6134 cohort. C, D) The correlations between the apCAFs and effector CD8⁺ T (CD8_Teff) cells in the TCGA-PDAC (C) and E-MTAB-6134 (D) cohort. E-I) The correlations between the iCAFs and Tregs (E), CD8_Tex (F), and CD4_Tex (G) cells, as well as between apCAFs and NKT (H) and CD8_Teff (I) cells in the PDAC_AU_array cohort. J-N) The correlations between the iCAFs and Tregs (J), CD8_Tex (K), and CD4_Tex (L) cells, as well as between apCAFs and NKT (M) and CD8_Teff (N) cells in the PDAC_AU_seq cohort. O-S) The correlations between the iCAFs and Tregs (O), CD8_Tex (P), and CD4_Tex (Q) cells, as well as between apCAFs and NKT (R) and CD8_Teff (S) cells in the PDAC_CA_seq cohort.


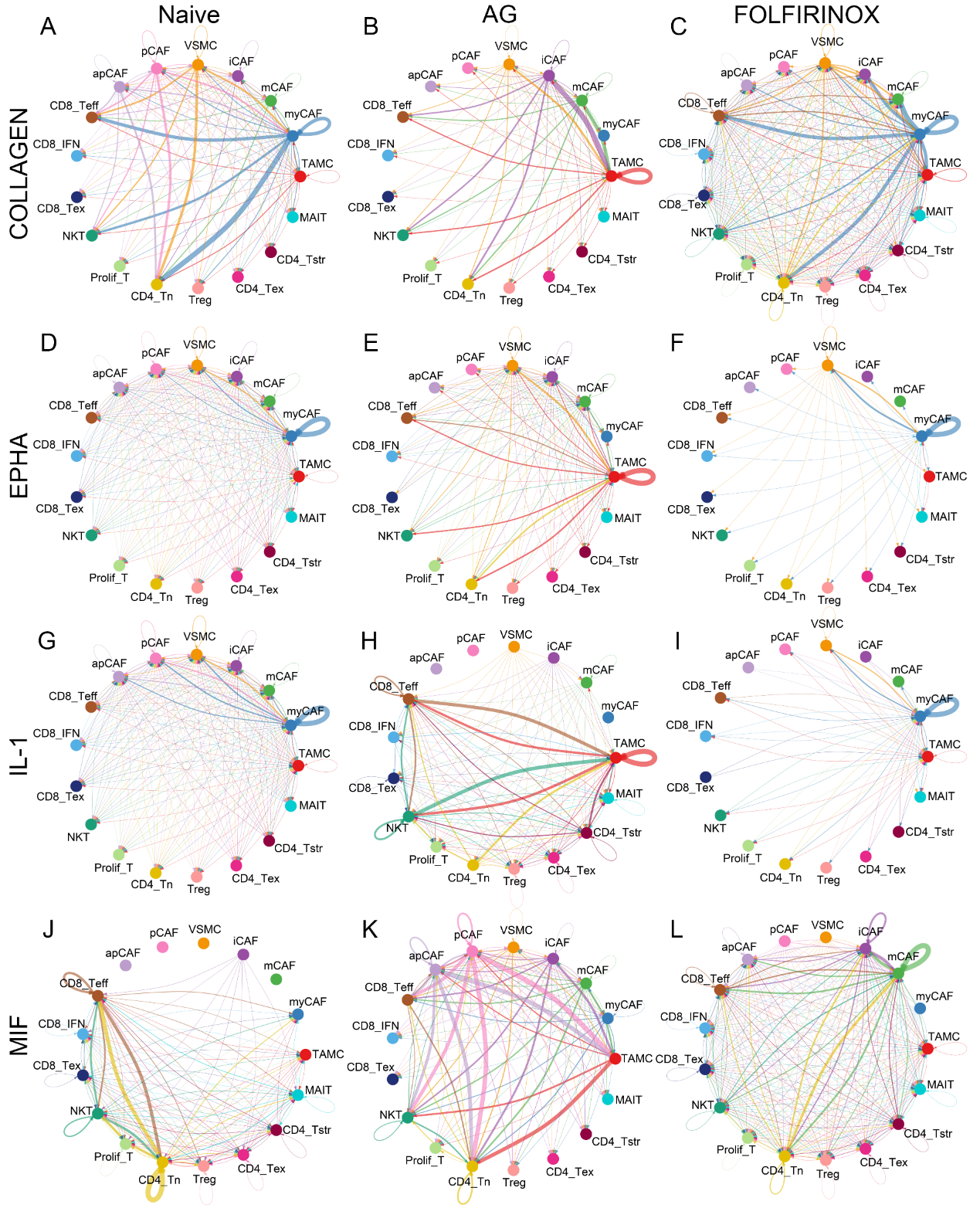


**Figure S12.** Cell-cell communication between TAMCs, six CAF subtypes, and 10 T-cell subtypes. A-C) Cell-cell communication networks on COLLAGEN signaling among tumor-associated mast cells (TAMCs), six cancer-associated fibroblast (CAF) subtypes, and 10 T-cell subsets in the naive (A), AG (B), and FOLFIRINOX (C) groups. D-F) Cell-cell communication networks on EPHA signaling among TAMCs, six CAF subtypes, and 10 T-cell subsets in the naive (D), AG (E), and FOLFIRINOX (F) groups.

G-I) Cell-cell communication networks on IL-1 signaling among TAMCs, six CAF subtypes, and 10 T-cell subsets in the naive (G), AG (H), and FOLFIRINOX (I) groups. J-L) Cell-cell communication networks on MIF signaling among TAMCs, six CAF subtypes, and 10 T-cell subsets in the naive (J), AG (K), and FOLFIRINOX (L) groups.


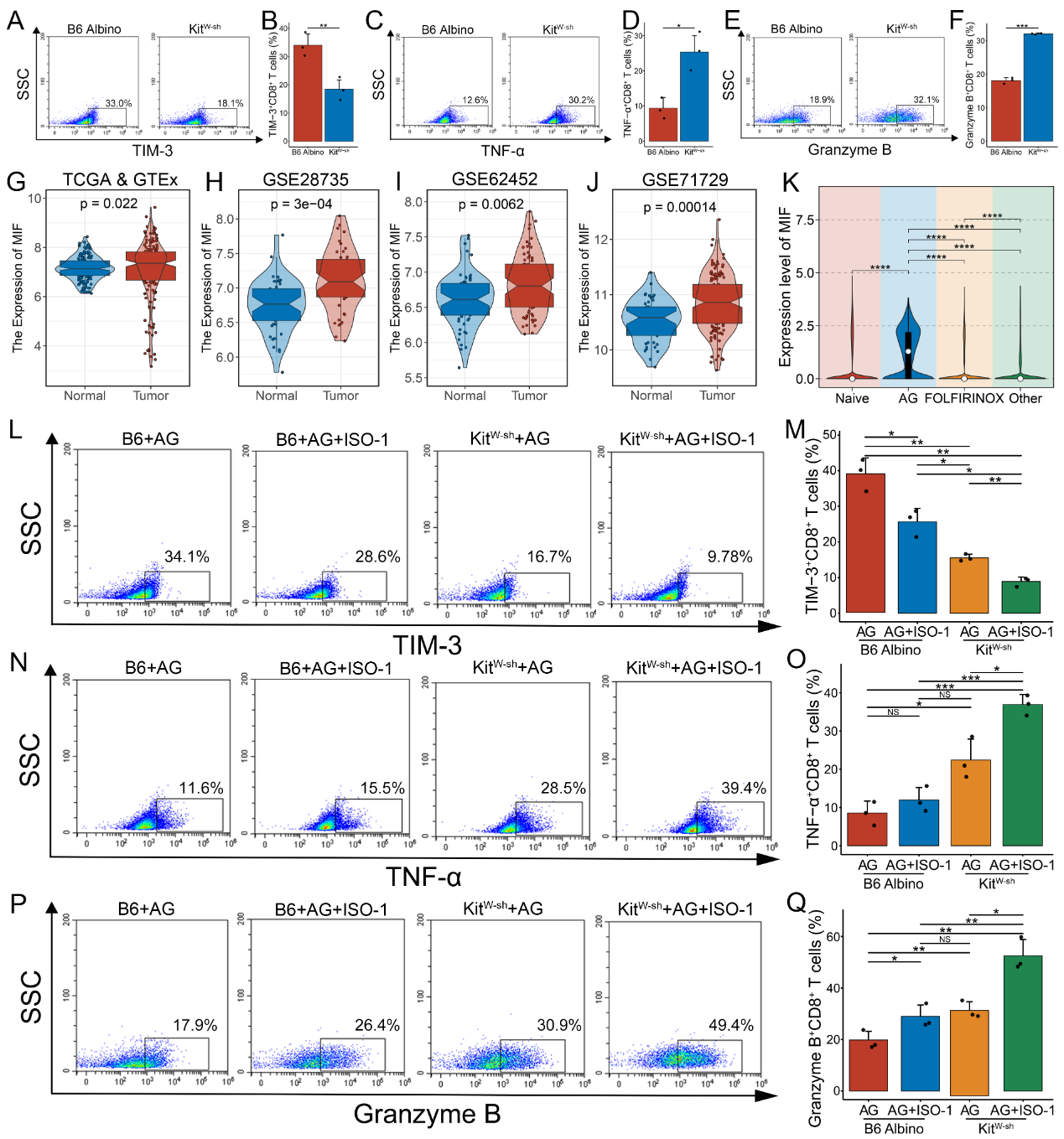


**Figure S13.** Experimental validation of targeting TAMCs and MIF signaling to reverse immunosuppressive microenvironment. A-F) Flow cytometry analysis of B6 Albino and Kit^W-sh^ genetically engineered mice showing the proportions of TIM-3^+^ exhausted CD8^+^ T cells (A, B) as well as TNF-α^+^ (C, D) and GZMB⁺ (E, F) effector CD8^+^ T cells. G-J) Expression levels of macrophage migration inhibitory factor (MIF) in tumor and normal samples from TCGA-PDAC and GTEx-Pancreas (G), GSE28735 (H), GSE62452 (I), and GSE71729 (J) cohorts. K) Violin plots showing observably up-regulated expression of MIF in cancer-associated fibroblasts (CAFs) in the AG neoadjuvant therapy groups. L-Q) Flow cytometry analysis of orthotopic tumors harvested from four treatment groups showing the proportions of TIM-3^+^ exhausted CD8^+^ T cells (L, M) as well as TNF-α^+^ (N, O) and GZMB⁺ (P, Q) effector CD8^+^ T cells.
